# Regulatory network for *FOREVER YOUNG FLOWER*-like genes in regulating *Arabidopsis* flower senescence and abscission

**DOI:** 10.1038/s42003-022-03629-w

**Published:** 2022-07-05

**Authors:** Wei-Han Chen, Pei-Tzu Lin, Wei-Han Hsu, Hsing-Fun Hsu, Ya-Chun Li, Chin-Wei Tsao, Mao-Cheng Hsu, Wan-Ting Mao, Chang-Hsien Yang

**Affiliations:** 1 Institute of Biotechnology, National Chung Hsing University, Taichung, Taiwan, 40227 ROC; 2 Advanced Plant Biotechnology Center, National Chung Hsing University, Taichung, Taiwan, 40227 ROC

**Keywords:** Plant molecular biology, Plant physiology

## Abstract

*FOREVER YOUNG FLOWER (FYF)* has been reported to play an important role in regulating flower senescence/abscission. Here, we functionally analyzed five *Arabidopsis FYF*-like genes, two in the *FYF* subgroup (*FYL1/AGL71* and *FYL2/AGL72*) and three in the *SOC1* subgroup (*SOC1/AGL20, AGL19,* and *AGL14*/*XAL2*), and showed their involvement in the regulation of flower senescence and/or abscission. We demonstrated that in *FYF* subgroup, *FYF* has both functions in suppressing flower senescence and abscission, *FYL1* only suppresses flower abscission and FYL2 has been converted as an activator to promote flower senescence. In *SOC1* subgroup, *AGL19*/*AGL14/SOC1* have only one function in suppressing flower senescence. We also found that FYF-like proteins can form heterotetrameric complexes with different combinations of A/E functional proteins (such as AGL6 and SEP1) and AGL15/18-like proteins to perform their functions. These findings greatly expand the current knowledge behind the multifunctional evolution of *FYF*-like genes and uncover their regulatory network in plants.

## Abstract (web summary)

Functional characterization of the FYF-like genes in Arabidopsis demonstrates roles in flower senescence/abscission and finds the FYF-associated heterotetrameric complexes in regulating flower abscission.

## Introduction

We have previously reported that *Arabidopsis FOREVER YOUNG FLOWER* (*FYF)* specifically regulates flower organ senescence and abscission by suppressing the downstream genes of ethylene signaling *EDF1/2/3/4* and abscission-associated genes *BOP1/2* and *IDA*^1,2^. Conserved functions in regulating flower organ senescence and abscission have been reported for *FYF* orthologs identified in different species of orchids^3–5^. Based on sequence homology and phylogenetic analysis, six putative closely related *FYF*-like genes could be subjected to two subgroups: (1) the *FYF* group composed of *FYF, AGL71*, and *AGL72* and (2) the *SOC1* group composed of *AGL20* (*SOC1), AGL19*, and *AGL14* (*XAL2*) in the *Arabidopsis* genome^6^.

It has been reported that additional new genes could be generated in the genome through gene duplication, which was thought to play an important role in organisms during evolution^7–9^. Phylogenetic analysis revealed that three duplication events from an *FYF*-like ancestor may have occurred (two within subgroups) to generate six *Arabidopsis FYF*-like genes^6^. Functional analysis indicated that the majority of the duplicate *FYF*-like gene pairs (*FYF/AGL71/AGL72/SOC1/AGL19/AGL14*) may retain overlap of the original ancestral function, such as the regulation of flowering time^10–23^, or have specific subsets of the original ancestral function, such as the regulation of root^24,25^ and ovule^26^ development as seen for *AGL14* (*XAL2*), which was described as subfunctionalization^9,27–30^. Although different putative functions were uncovered for these *Arabidopsis FYF*-like genes, exploration of the function in regulating flower organ senescence and abscission was investigated for only the *FYF* gene and has never been reported for other five *Arabidopsis FYF*-like genes. It is therefore not clear whether the *FYF* gene evolved to have this unique function in regulating flower organ senescence and abscission from its ancestor or whether some of the other *FYF*-like genes also harbored this function during evolution. To uncover these questions, we comprehensively functionally characterized all putative *Arabidopsis FYF*-like genes for their involvement in regulating flower organ senescence and abscission. Furthermore, we used a FRET-based strategy to investigate the possible heterotetrameric protein complexes formed by the interactions of FYF-like and other MADS box proteins to further verify the regulatory networks for these duplicate *FYF*-like gene pairs in *Arabidopsis*.

Here, we show that all other five *Arabidopsis FYF*-like genes have a function in regulating flower senescence and/or abscission similar to *FYF*. In this work, we also found that FYF-like proteins can interact with different combinations of A/E functional proteins (such as AGL6 and SEP1) and AGL15/18-like proteins (such as AGL15/18) to form heterotetrameric complexes in regulating flower senescence and abscission.

## Results

### Characterization of two *FYF* closely related genes, *FYL1* and *FYL2*

Two *Arabidopsis thaliana* MADS box genes, known to be closely related to *FYF* (AT5G62165), named *FYF-like 1, 2 (FYL1/AGL71, FYL2/AGL72)*, in the *FYF* group were analyzed. *FYL1* (AT5G51870*)* encodes a protein containing 219 amino acids that showed 40.3% identity to *FYF*, with 86.2% (50/58) of the amino acids being identical in the MADS box domain (Supplementary Fig. 1). *FYL2* (AT5G51860*)* encodes a protein containing 202 amino acids that showed 43.7 and 65% identity to *FYF* and *FYL1*, respectively, with 86.2% (50/58) and 94.8% (55/58) of the amino acids being identical in the MADS box domain (Supplementary Fig. 1), respectively. The amino acid identity and the phylogenetic tree relationship^6^ indicated that FYL1 was more closely related to FYL2 than to FYF (Supplementary Fig. 2).

### The distinct expression patterns of *FYL1* and *FYL2*

**Fig. 1:**
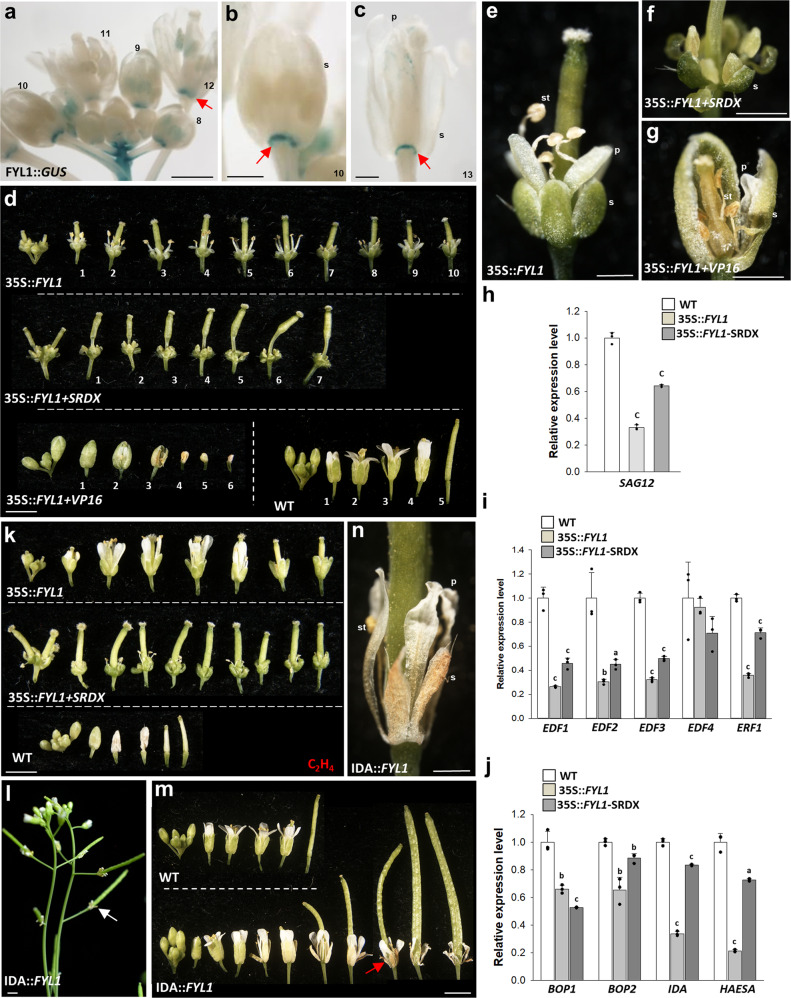
Characterization of the *FYL1* gene through transgenic plants and gene expression analysis in *Arabidopsis*. **a** GUS was strongly stained in the AZ (arrowed) of floral buds and mature flowers of FYL1::*GUS Arabidopsis*. The numbers indicate the different developmental stages of *Arabidopsis* flowers. Bar = 1 mm. Magnified view of stage 10 (**b**) and 13 (**c**) FYL1::*GUS* flowers. GUS was only strongly stained in the AZ (arrowed). s: sepal, p: petal. Bars = 0.5 mm. **d** Flowers along the inflorescences of 35S::*FYL1* (first row), 35S::*FYL1*+*SRDX* (second row) and 35S::*FYL1*+*VP16* (third row, left) and wild-type (WT) (third row, right) plants. The numbers indicate the positions of the flowers. Bar = 1.5 mm. Magnified view of the delayed senescent 35 S::*FYL1* (**e**), 35S::*FYL1*+*SRDX* (**f**) and early senescent 35S::*FYL1*+*VP16* (**g**) flowers. s: sepal, p: petal, st: stamen. Bars = 0.5 mm. Detection of *SAG12* (**h**), *EDF1-4* and *ERF1* (**i**) and *BOP1/2, IDA* and *HAESA* (**j**) expression in 35S::*FYL1* and 35S::*FYL1*+*SRDX Arabidopsis*. Error bars show ± SD. *n* = 3 biologically independent samples. The expression of each gene in the transgenic plants is given relative to that of the wild-type plant, which was set at 1. The letter “a”, “b” and “c” indicates significant difference from the wild-type (WT) value (a: *P* < 0.05, b: *P* < 0.01, and c: *P* < 0.001). The two-sided Student’s *t*-test was used. **k** Flowers along the inflorescence of 35S::*FYL1* (first row), 35S::*FYL1* + *SRDX* (second row), and wild-type (third row) plants after exposure to ethylene. Bar = 2 mm. Inflorescence of IDA::*FYL1* plants (**l**) and flowers along the inflorescence (**m**) of IDA::*FYL1* (bottom) and wild-type (WT, top) plants. The flower organs (arrowed) remained on the IDA::*FYL1* flower and siliques. Bars = 2 mm. **n** Magnified view of an IDA::*FYL1* flower with a senescent but not abscised phenotype from (**m**). s: sepal, p: petal, st: stamen. Bar = 0.5 mm.

**Fig. 2:**
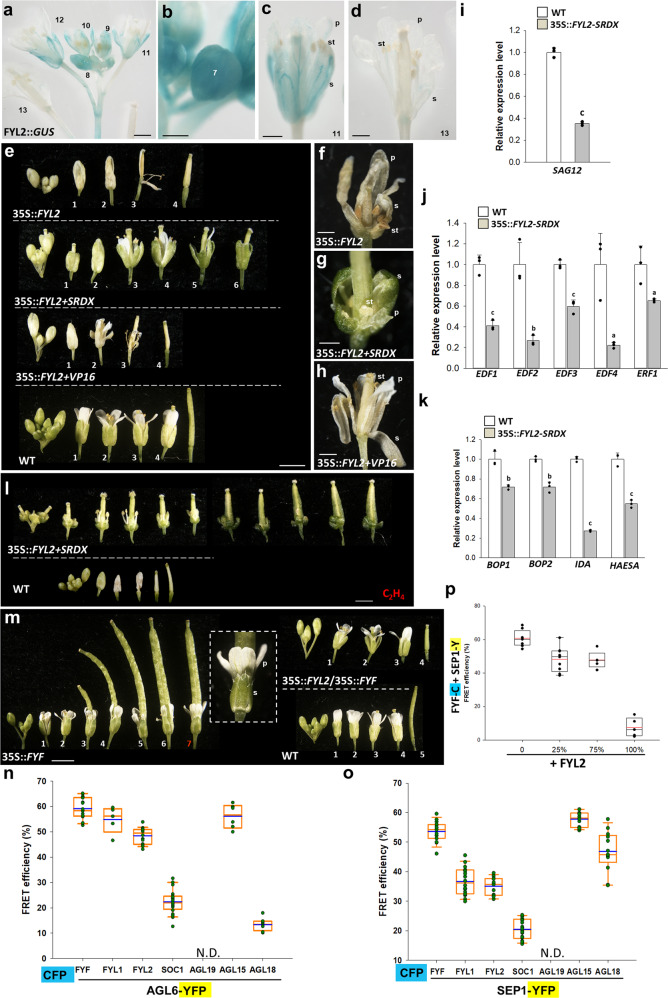
Characterization of the *FYL2* gene through transgenic plants and gene expression analysis in *Arabidopsis*. **a** GUS was stained in the sepals/petals of flowers of FYL2::*GUS Arabidopsis*. GUS staining gradually decreased in the mature flowers during the late stage of flower development. The numbers indicate the different developmental stages of *Arabidopsis* flowers. Bar =1 mm. Magnified view of stage 7 (**b**), 11 (**c**), and 13 (**d**) FYL2::*GUS* flowers. GUS was strongly and relatively weakly stained in stage 7 young and 11 mature flower buds and was barely detected in stage 13 mature flowers. s: sepal, p: petal, st: stamen. Bars = 0,5 mm. **e** Flowers along the inflorescences of 35S::*FYL2* (first row), 35S::*FYL2*+*SRDX* (second row), 35S::*FYL2*+*VP16* (third row) and wild-type (WT) (fourth row) plants. The numbers indicate the positions of the flowers. Bar = 2 mm. Magnified view of early senescent 35S::*FYL2* (**f**) and 35S::*FYL2*+*VP16* (**h**) flowers and delayed senescent 35S::*FYL2*+*SRDX* (**g**) flowers. s: sepal, p: petal, st: stamen. Bars = 0.5 mm. Detection of *SAG12* (**i**), *EDF1-4* and *ERF1* (**j**) and *BOP1/2, IDA* and *HAESA* (**k**) expression in 35S::*FYL2*+*SRDX Arabidopsis*. Error bars show ± SD. *n* = 3 biologically independent samples. The expression of each gene in the transgenic plants is given relative to that of the wild-type plant, which was set at 1. The letter “a”, “b” and “c” indicates significant difference from the wild-type (WT) value (a: *P* < 0.05, b: *P* < 0.01, and c: *P* < 0.001). The two-sided Student’s *t*-test was used. **l** Flowers along the inflorescence of 35S::*FYL2*+*SRDX* (top) and wild-type (bottom) plants after exposure to ethylene. Bar = 1 mm. **m** Flowers along the inflorescence of 35S::*FYF*, 35S::*FYF/*35S::*FYL2,* and wild-type (WT) plants. Magnified view of the flower organs that did not become senescent and did not undergo abscission (boxed) in the #7 35 S::*FYF* flower. s: sepal, p: petal. Bar = 2 mm. Analysis of the interaction of MADS proteins FYF, FYL1, FYL2, SOC1, AGL19, AGL15, and AGL18 fused with CFP to AGL6 (**n**) and SEP1 (**o**) fused with YFP through the FRET technique. CyPet- and YPet-fused protein pair fluorescence signals were detected in the nucleus expressed in tobacco leaves. CFP and YFP channels were excited with a 440 nm laser, and these two channels were used to calculate the raw FRET signal. FRET values were divided by CFP signals to calculate the FRET efficiency. The average FRET efficiency values were quantified in multiple samples (*n* > 4). Image frame = 20 × 20 µm^2^. N.D. indicates not determined. (Blue line: mean). **p** Analysis of the effect of FYL2 on FYF-SEP1 interactions. The FRET efficiency for the formation of FYF-CFP/SEP1-YFP complexes was analyzed in tobacco cells by adding different amounts (0, 25, 75, and 100%) of unlabeled FYL2 proteins. The average FRET efficiency values were quantified in multiple samples (*n* > 4). Image frame = 20 × 20 µm^2^. (Red line: mean).

To investigate the expression patterns of the *FYL1* and *FYL2* genes, *FYL1/2* expression was further analyzed in flowers at different developmental stages. The results indicated that higher *FYL1/2* expression was observed during early flower development (before stage 9) than during late developmental stages (after stage 12) (Supplementary Fig. 3a, b), which was similar to the spatial and temporal expression pattern of *FYF* during flower development (Supplementary Fig. 3c)^1^. When FYL1::*GUS* and FYL2::*GUS* constructs were generated and transformed into *Arabidopsis*, GUS staining was exclusively detected in the abscission zone (AZ) of the sepals/petals of FYL1::*GUS* flowers (Fig. 1a–c) and shows a more extended pattern in the sepals/petals of FYL2::*GUS* flowers (Fig. 2a–d). Further RT-qPCR analysis indicated that *FYL1* expression was highly detected whereas *FYL2* expression was almost undetectable in the AZ (Supplementary Fig. 3d). This result is interesting since GUS staining was detected in both sepals/petals and in the abscission zones of FYF::*GUS* flowers^1^, suggesting that *FYL1* and *FYL*2 might have different subfunctions of *FYF* in regulating flower senescence and abscission.

### *FYL1* delayed flower senescence and abscission once ectopically expressed in transgenic *Arabidopsis* plants

To investigate function correlated to the expression pattern of *FYL1*, 35S::*FYL1*, 35S::*FYL1*+*SRDX* (containing a suppression motif), and 35S::*FYL1-DR*+*VP16* (containing an activation VP16-AD) were transformed into *Arabidopsis*. The 35S::*FYL1* plants showed a delay in both flower senescence and abscission (Fig. 1d, top and 1e), similar to what was observed in 35S::*FYF* plants^1^. Furthermore, enhancement of the delay of flower senescence/abscission was observed in 35S::*FYL1*+*SRDX* transgenic plants (Fig. 1d, middle and 1f), and an opposite promotion of flower senescence/abscission was produced in 35S::*FYL1-DR*+*VP16* transgenic plants (Fig. 1d, bottom left and 1g), suggesting that FYL1 should act as a repressor in suppressing flower senescence/abscission, similar to FYF. Once it has been converted into an activator, *FYL1-DR*+*VP16*, an opposite dominant negative mutant phenotype will be observed. We also found that the expression of the senescence-associated gene *SAG12*, downstream genes in ethylene signaling *EDF1-3*, and *ERF1* and abscission-associated genes *BOP1/2, IDA*, and *HAESA* were all downregulated in 35S::*FYL1* and 35S::*FYL1*+*SRDX* plants (Fig. 1h–j). In addition, 35S::*FYL1* and 35S::*FYL1*+*SRDX* flowers were insensitive to ethylene treatment (Fig. 1k). Our data suggest that *FYL1* could have a prominent role like *FYF* in controlling floral senescence/abscission once ectopically expressed in *Arabidopsis* flowers. However, *FYL1* should only have a partial role in controlling floral abscission in real life since its expression was restricted to the abscission zone (AZ) of the sepals/petals of flowers (Fig. 1a–c and Supplementary Fig. 3d).

To further confirm the relationship between *FYL1* and sepal/petal abscission, the *FYL1* gene driven by the *IDA* promoter (IDA::*FYL1*) was transformed into *Arabidopsis*. A clear delay in the abscission of the perianth organs was observed in IDA::*FYL1* flowers (Fig. 1l–n). However, senescence of the perianth normally occurred from positions 4–5 in these flowers that were not abscised in the *IDA:FYL1* transgenic plants (Fig. 1l–n). This phenotype was very similar to what has been observed in *ida* mutants^31^ and IDA::*FYF* plants^1^; thus, the result supported the role of FYL1 as a suppressor and its function together with FYF in suppressing *IDA* and sepal/petal abscission.

### *FYL2* promoted flower senescence and abscission when ectopically expressed in transgenic *Arabidopsis* plants

Similar to *FYL1*, 35S::*FYL2*, 35S::*FYL2*+*SRDX,* and 35S::*FYL2-DR*+*VP16 Arabidopsis* were generated. 35S::*FYL2* plants surprisingly showed promotion of both flower senescence and abscission (Fig. 2e, first row and 2f). A similar promotion of flower senescence/abscission was observed in 35S::*FYL2-DR*+*VP16* transgenic plants (Fig. 2e, third row and 2h), and an opposite delay of flower senescence/abscission was produced in 35S::*FYL2*+*SRDX* transgenic plants (Fig. 2e, second row and 2g), suggesting that FYL2 should act as an activator in promoting flower senescence/abscission, in contrast to FYF and FYL1. We also found that the expression of *EDF1-4, ERF1*, *BOP1/2, IDA*, and *HAESA* were all downregulated in 35S::*FYL2*+*SRDX* plants (Fig. 2i–k). By contrast, *SAG12*, *EDF1-4, ERF1, IDA*, and *HAESA* were all upregulated in 35S::*FYL2* and 35S::*FYL2-DR*+*VP16* transgenic plants (Supplementary Fig. 4). In addition, 35S::*FYL2*+*SRDX* flowers were insensitive to ethylene treatment (Fig. 2l). This result suggested that *FYL2* should function opposite to FYF and could have the same role as *FYF* once it is converted into a repressor as *FYL2*+*SRDX*, and a dominant negative mutant phenotype in suppressing floral senescence/abscission will be observed. However, *FYL2* should only have an opposite role to *FYF* in controlling floral senescence in real life since its expression was specifically in the sepals/petals of flowers (Fig. 2a–d).

To further confirm the relationship between *FYL2* and *FYF* in regulating sepal/petal senescence, 35S::*FYF* and 35S::*FYL2* were doubly transformed into *Arabidopsis*, and plants ectopically expressing *FYF* and FYL2 were generated simultaneously. A clear wild-type-like phenotype by senescence and abscission of the perianth organs at approximately positions 3–4 was observed in the 35S::*FYF*/35S::*FYL2* flowers (Fig. 2m, right), which was earlier than that in the 35S::*FYF* flowers (Fig. 2m, left) and later than that in the 35S::*FYL2* flowers (Fig. 2e, first row). This 35S::*FYF*/35S::*FYL2* intermediate phenotype between 35S::*FYF* and 35S::*FYL2* clearly indicated an antagonistic relationship between *FYF* and *FYL2*. Thus, the results supported a role for FYL2 as an activator with a function antagonistic to part of the FYF function in controlling senescence of the sepal/petal.

### FYF can interact with AGL6 and SEP1 in regulating flower abscission/senescence

To investigate which proteins could possibly interact with FYF to form a complex in regulating flower organ abscission and senescence, two potential candidates, AGL6 and SEP1, which have been reported to be able to interact with FYF through yeast two-hybrid screen^32^, were identified. It is important to determine whether the spatial and temporal expression patterns of the *AGL6* and *SEP1* genes were correlated with *FYF*. Based on the AGL6::*GUS* assay, *AGL6* expression could be detected in the basal parts and abscission zone of the floral organs during floral development (Supplementary Fig. 5a–c)^33^ and was detected to be more abundant in wild-type flowers before (BP) than after (AP) pollination (Supplementary Fig. 5d), which overlapped with the expression pattern of *FYF*. *SEP1* has been reported to be expressed in all four whorls of flower organs, is more abundant during early morphological differentiation than in mature flowers, and was not reported in the abscission zone during floral development^34,35^. These results suggested that FYF might interact with AGL6 to form a complex in regulating sepal/petal abscission. In addition, FYF can interact separately with SEP1 and AGL6 to form a complex in regulating sepal/petal senescence.

**Fig. 3:**
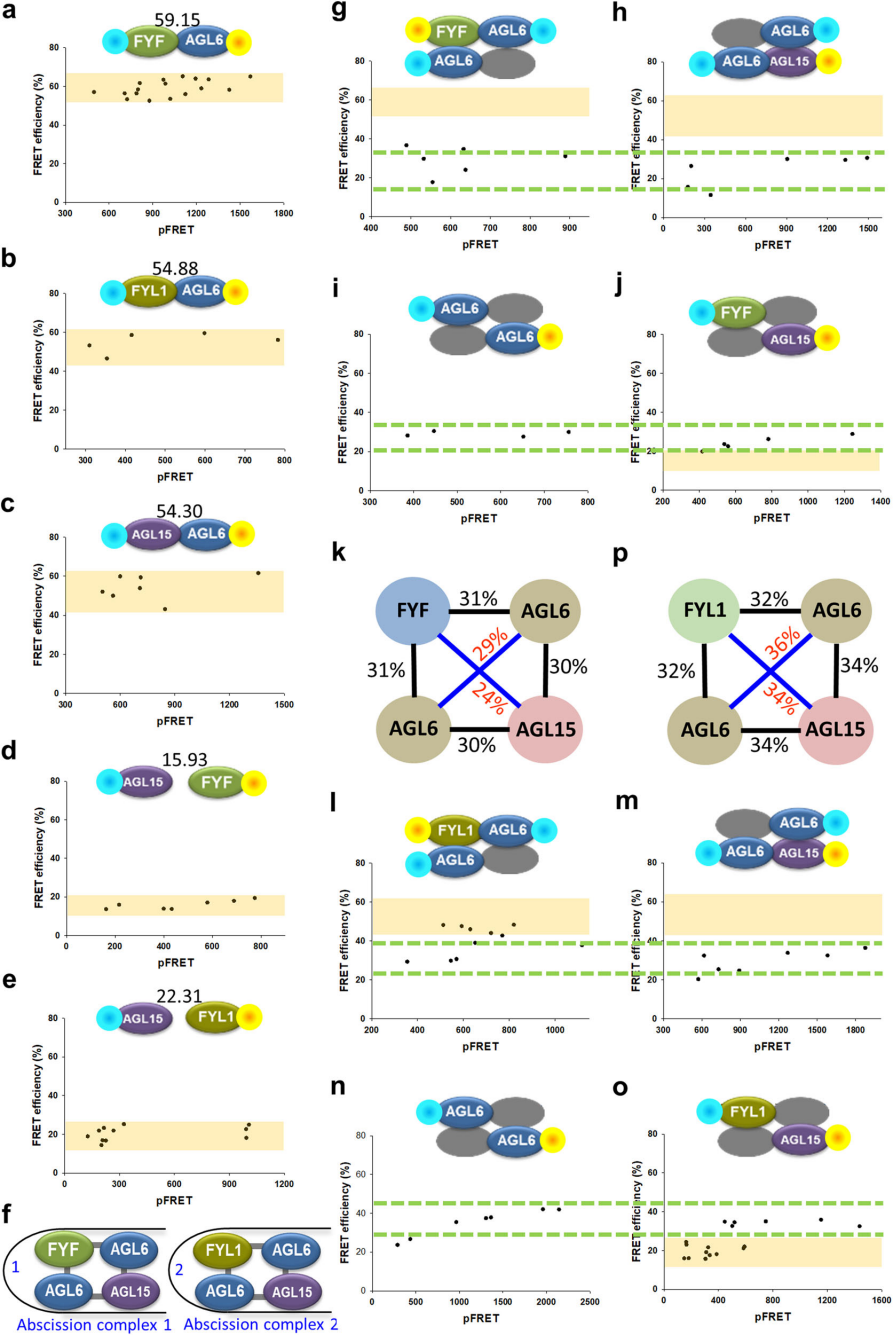
The distance-measuring system validated that *Arabidopsis* AGL15-AGL6 forms stable abscission tetrameric complexes with FYF-AGL6 and FYL1-AGL6. The steady state of dimerization of the protein complexes FYF-AGL6 (**a**), FYL1-AGL6 (**b**), AGL15-AGL6 (**c**), FYF-AGL15 (**d**), and FYL1-AGL15 (**e**) is revealed in scatter diagrams showing pFRET and FRET efficiency. The black dots show the independent cell nuclei, and the yellow boxes indicate the steady-state FRET efficiency range for the protein complex. The mean value of FRET efficiency in the steady state is shown at the top of the schematic model, which is the baseline. The protein fused with CFP/YFP was attached by blue/yellow spots. **f** Schematic model of the protein interactions in the *Arabidopsis* abscission complexes (1) FYF-AGL6-AGL6-AGL15 and (2) FYL1-AGL6-AGL6-AGL15. Scatter diagram of the raw FRET (pFRET) and FRET efficiency values of the dimer pairs in adjacent lines AGL6-FYF (**g**) and AGL6-AGL15 (**h**) and diagonal lines AGL6-AGL6 (**i**) and FYF-AGL15 (**j**) in the abscission complex FYF-AGL6-AGL6-AGL15, with a different number of cell nuclei measured. The green dotted lines indicate the overlapping distribution range at the steady state. The yellow boxes (in **g**, **h**, **j**) indicate the baselines obtained for the dimer pairs. The protein fused with CFP/YFP was attached by blue/yellow spots. **k** Schematic model and FRET efficiency of four different pairs (two adjacent lines and two diagonal lines) of the protein interactions in the *Arabidopsis* stable abscission complex FYF-AGL6-AGL6-AGL15. The two adjacent lines (black) show similar FRET efficiencies (31%/30%), and the two diagonal lines (blue) show similar FRET efficiencies (29%/24%). Scatter diagram of the raw FRET (pFRET) and FRET efficiency values of the dimer pairs in adjacent lines AGL6-FYL1 (**l**) and AGL6-AGL15 (**m**) and diagonal lines AGL6-AGL6 (**n**) and FYL1-AGL15 (**o**) in the abscission complex FYL1-AGL6-AGL6-AGL15, with a different number of cell nuclei measured. The green dotted lines indicate the overlapping distribution range at the steady state. The yellow boxes (in **l**, **m**, **o**) indicate the baselines obtained for the dimer pairs. The protein fused with CFP/YFP was attached by blue/yellow spots. **p** Schematic model and FRET efficiency of four different pairs (two adjacent lines and two diagonal lines) of the protein interactions in the *Arabidopsis* stable abscission complex FYL1-AGL6-AGL6-AGL15. The two adjacent lines (black) show similar FRET efficiencies (32%/32%), and the two diagonal lines (blue) show similar FRET efficiencies (36%/34%).

We further performed FRET analyses by using FYF-CFP and AGL6/SEP1-YFP to observe physical interactions of FYF and AGL6/SEP1 protein complexes in tobacco cells^36^. The results confirmed that FYF/AGL6 formed heterodimers with high efficiency in the nucleus (Fig. 2n, column 1, Fig. 3a). The same result was observed for FYF/SEP1 (Fig. 2o, column 1). These results suggested that FYF is able to interact with AGL6 and SEP1 to form complexes in regulating flower senescence and/or abscission.

### FYL1 can interact with AGL6 in regulating flower abscission

To further investigate whether FYL1 could also interact with proteins similar to FYF in regulating flower organ abscission, FRET analyses were performed. When FYL1-CFP and AGL6/SEP1-YFP were used to observe the physical interactions of FYL1 and AGL6/SEP1, FYL1/AGL6 can form heterodimers with similar efficiency to FYF/AGL6 in the nucleus of tobacco cells (Fig. 2n, column 2, Fig. 3b). However, the efficiency for the formation of FYL1/SEP1 (Fig. 2o, column 2) was clearly lower than that for FYF/SEP1 (Fig. 2o, column 1). These results suggested that FYL1 is able to interact with AGL6 in a more stable manner than with SEP1 to form complexes. Since *FYL1* was only expressed in the AZ of the sepals/petals and could only regulate flower organ abscission, it is reasonable to believe that *FYL1* can only physically interact with AGL6 in the AZ to regulate abscission of the sepals/petals. Although they can interact, the *FYL1/SEP1* complex should not exist during *Arabidopsis* flower development since these two genes have no overlapping expression pattern.

The ability of AGL6 to interact with FYF and FYL1 to form complexes reveals that its function should be related to *FYF/FYL1*. This assumption was further supported by the result that a similar delay in the flower senescence/abscission phenotype (Supplementary Fig. 5e–i) and downregulation of *EDF1, BOP1/2, IDA*, and *HAESA* (Supplementary Fig. 5j, k) were observed in 35S::*AGL6*+*SRDX* plants. This result revealed that FYF/FYL1 can interact with AGL6 to target similar downstream genes once expressed in the same places.

### FYF and FYL1 can interact with AGL6/AGL6 and AGL15 proteins to form stable heterotetrameric abscission complexes

Based on the floral quartet model in which plant MADS-box proteins function as higher-order tetrameric complexes^37^, we hypothesized that FYF-AGL6 heterodimer proteins would further form heterotetrameric complexes with other MADS box proteins in the AZ to regulate sepal/petal abscission. It is interesting to note that two MADS box genes, *AGL15* and *AGL18*, have been reported to be expressed in flower organs and in the AZ of flowers^38,39^ (Supplementary Fig. 6a–d), and a delay in senescence/abscission of flowers has also been observed in 35S::*AGL15* and 35S::*AGL18 Arabidopsis* plants^38,39^. The similar delay of flower senescence/abscission (Supplementary Fig. 6e–h) and downregulation of *EDF1-4, ERF1. BOP1/2, IDA*, and *HAESA* (Supplementary Fig. 6i–j) in 35S::*AGL15*+*SRDX Arabidopsis* supports the notion that AGL15/AGL18 function similarly to FYF/FYL1 as repressors^39^ in regulating flower organ abscission. To further explore whether AGL15/AGL18 could also interact with proteins similar to FYF/FYL1 in regulating flower organ abscission, FRET analyses were performed. The results indicated that AGL15/AGL6 are able to form heterodimers with similar efficiency to FYF/AGL6 in the nucleus of tobacco cells (Fig. 2n, column 6, Fig. 3c). In contrast, AGL18 showed a very weak interaction with AGL6 (Fig. 2n, column 7). This result revealed that AGL15 can form a complex with AGL6, whereas AGL18 might form different protein complexes to perform the redundant function in regulating flower organ abscission.

To test whether FYF (FYL1), AGL6, and AGL15 could form heterotetrameric abscission complexes, a strategy using in vivo FRET based on the distance change and distance symmetry of a stable tetrameric complex in tobacco leaf cells was performed^40^. Since FYF is unlikely to interact with AGL15 (Fig. 3d) in the FRET analysis, we analyzed the possible abscission tetramer containing FYF/AGL6 and AGL15/AGL6 heterodimers. In the FYF-AGL6-AGL6-AGL15 complex (Fig. 3f–l), the FRET efficiency of the coexpression admixture of AGL6:CFP/FYF:YFP/AGL15 (Fig. 3g) was similar to that of AGL6:CFP/AGL15:YFP/FYF (Fig. 3h) (31%/30%) (Fig. 3k), and the distribution range overlapped. The FRET efficiency of the coexpression admixture of AGL6:CFP/AGL6:YFP/FYF/AGL15 (Fig. 3i) was similar to that of FYF:CFP/AGL15:YFP/AGL6/AGL6 (Fig. 3j) (29%/24%) (Fig. 3k), and the distribution range overlapped.

Similarly, FYL1 was unlikely to interact with AGL15 (Fig. 3e), and the FRET efficiency of the coexpression admixture of AGL6:CFP/FYL1:YFP/AGL15 (Fig. 3l) was similar to that of AGL6:CFP/AGL15:YFP/FYL1 (Fig. 3m) (32%/34%) (Fig. 3p), and the distribution range overlapped. Similarly, the FRET efficiency of the coexpression admixture of AGL6:CFP/AGL6:YFP/FYL1/AGL15 (Fig. 3n) was similar to that of AGL15:CFP/FYL1:YFP/AGL6/AGL6 (Fig. 3o) (36%/34%) (Fig. 3p), and the distribution range overlapped. These overlapped patterns for the distribution range observed for FYF-AGL6-AGL6-AGL15 and FYL1-AGL6-AGL6-AGL15 heterotetrameric complexes were similar to that for the most stable heterotetrameric complexes PI-AP3-AG-SEP3 described in our previous study^40^. These results indicated that FYF-AGL6-AGL6-AGL15 and FYL1-AGL6-AGL6-AGL15 are likely stable heterotetrameric complexes in regulating flower organ abscission. In addition, FYF-AGL6-AGL6-AGL15 is also likely a stable heterotetrameric complex in regulating flower organ senescence.

### FYL2 can interact with AGL6 and SEP1 in regulating flower senescence

Similarly, the investigation of whether FYL2 could also interact with proteins similar to FYF in regulating flower organ senescence was also performed using FRET analyses. When FYL2-CFP and AGL6-YFP or SEP1-YFP were used to observe the physical interactions of FYL2 and AGL6 or SEP1, a lower efficiency of FYL2/AGL6 heterodimer formation than that of FYF/AGL6 (Fig. 2n, column 1) in the nucleus of tobacco cells was observed (Fig. 2n, column 3). The efficiency for the formation of FYL2/SEP1 (Fig. 2o, column 3) was also lower than that for FYF/SEP1 (Fig. 2o, column 1). These results suggested that similar to FYF, FYL2 can also interact with AGL6 and SEP1 to form senescence complexes, although at a lower efficiency. Since *FYL2* was only expressed in the flower organs of sepals/petals, which overlapped with part of *AGL6* and *SEP1* expression, these data revealed that FYL2 can physically interact with AGL6 and SEP1 during *Arabidopsis* flower development to regulate sepal/petal senescence.

Since we have already shown that FYL2 functions opposite to FYF in controlling sepal/petal senescence, FYL2 might compete to bind the interacting protein to form a functional complex. To examine this assumption, FRET efficiency for the formation of FYF-CFP/SEP1-YFP complexes was examined in tobacco cells by adding different amounts of unlabeled FYL2 proteins. The results indicated that the efficiency for FYF-CFP to interact with SEP1-YFP (Fig. 2p, column 1) was clearly decreased by the presence of 25–75% of the FYL2 proteins (Fig. 2p, columns 2 and 3). The ability of FYF-CFP to interact with SEP1-YFP was almost completely competed for by the presence of 100% FYL2 protein (Fig. 2p, column 4). Thus, FYL2 competes with FYF to interact with SEP1, performing opposite functions in controlling sepal/petal senescence.

### *FYF*-like genes *AGL19/14* and *SOC1* are complementary to *FYF* in regulating flower senescence

**Fig. 4:**
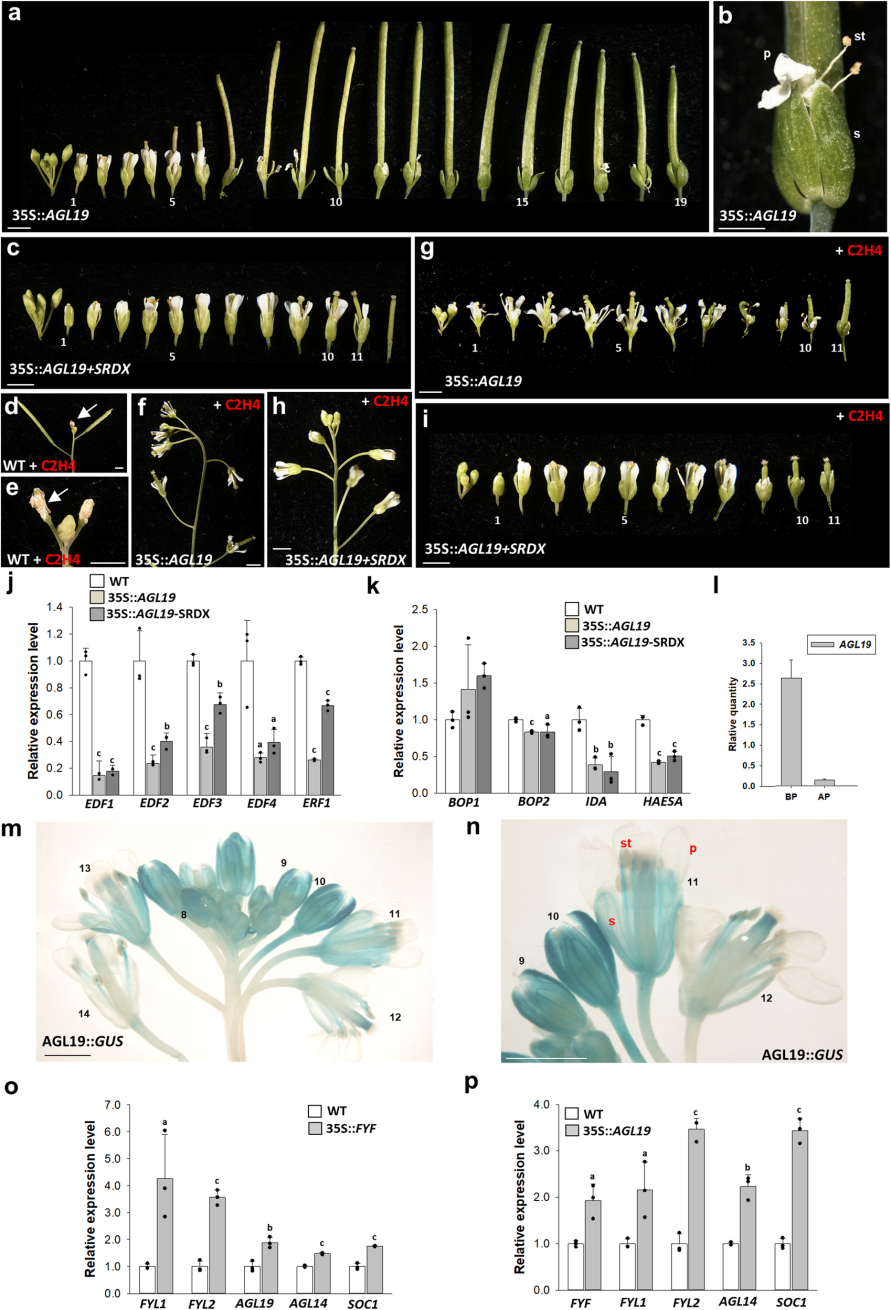
Characterization of the *AGL19* gene through transgenic plants and gene expression analysis in *Arabidopsis*. **a** Flowers along the inflorescence of 35S::*AGL19* plants. The numbers indicate the positions of the flowers. Bars = 2 mm. **b** Magnified view of the flower organs of a 35S::*AGL19* flower that were not senescent/abscised from (**a**). s: sepal, p: petal, st: stamen. Bars = 0.5 mm. **c** Flowers along the inflorescence of 35S::*AGL19*+*SRDX* plants. The numbers indicate the positions of the flowers. Bars = 2 mm. Flowers along the inflorescence of wild-type (**d**, **e**), 35S::*AGL19* (**f**, **g**), and 35S::*AGL19*+*SRDX* (**h**, **i**) plants after exposure to ethylene. Wild-type flowers were senescent (arrowed in **d**, **e**), whereas 35S::*AGL19* and 35S::*AGL19*+*SRDX* flowers were not senescent/abscised. Bars = 2 mm. Detection of *EDF1–4* and *ERF1* (**j**) and *BOP1/2, IDA* and *HAESA* (**k**) expression in 35S::*AGL19* and 35S::*AGL19*+*SRDX Arabidopsis*. Error bars show ± SD. *n* = 3 biologically independent samples. The expression of each gene in the transgenic plants is given relative to that of the wild-type plant, which was set at 1. The letter “a”, “b” and “c” indicates significant difference from the wild-type (WT) value (a: *P* < 0.05, b: *P* < 0.01, and c: *P* < 0.001). The two-sided Student’s *t*-test was used. **l** Detection of *AGL19* expression before (BP) and after (AF) pollination. **m**, **n** GUS was stained in the sepals/petals of flowers of AGL19::*GUS Arabidopsis*. GUS was strongly stained in stage 8–10 young flower buds and gradually decreased in the mature flowers during the late stage (after stage 12) of flower development. The numbers indicate the different developmental stages of *Arabidopsis* flowers. **n** is the magnified view from (**m**). s: sepal, p: petal, st: stamen. Bars = 1 mm. Detection of *FYL1/FYL2/AGL19/AGL14/SOC1* expression in 35S::*FYF Arabidopsis* (**o**) and *FYF/FYL1/FYL2/AGL14/SOC1* expression in 35S::*AGL19 Arabidopsis* (**p**). Error bars show ± SD. *n* = 3 biologically independent samples. The expression of each gene in the transgenic plants is given relative to that of the wild-type plant, which was set at 1. The letter “a”, “b” and “c” indicates significant difference from the wild-type (WT) value (a: *P* < 0.05, b: *P* < 0.01, and c: *P* < 0.001). The two-sided Student’s *t*-test was used.

We found a possible mechanism involving three *FYF*-like genes (*FYF* and *FYL1/2*) in regulating flower organ senescence and abscission. It is interesting to note that three other genes in the *SOC1* subgroup, *AGL19, AGL14 (XAL2),* and *AGL20 (SOC1)*, were also closely related to the *FYF/FYL1/FYL2* genes (Supplementary Figs. 1, 2)^6,16^. Do these three genes also harbor similar functions to *FYF/FYL1/FYL2* in regulating flower senescence/abscission? Interestingly, similar to that observed in 35S::*FYF* and 35S::*FYF*+*SRDX Arabidopsis*, a strong delay in flower senescence and abscission (Fig. 4a–c), insensitivity to ethylene treatment (Fig. 4d–i) and downregulation of *EDF1-4, ERF1*, *BOP2, IDA*, and *HAESA* (Fig. 4j, k) were observed in 35S::*AGL19* and 35S::*AGL19*+*SRDX Arabidopsis*. In contrast to *AGL19*, only 35S::*AGL14*+*SRDX* and 35S::*SOC1*+*SRDX* caused a strong delay in flower senescence/abscission and a downregulation of senescence/abscission-related genes (Supplementary Figs. 7a–d, 8a–c), whereas no or a reduced effect was seen in 35S::*AGL14* and 35S::*SOC1* plants. These results indicated that *AGL19* and *AGL14/SOC1* functioned as strong and weak repressors, respectively, and that part of their function complemented *FYF* in suppressing flower organ senescence/abscission.

Similar to the expression pattern of *FYF/FYL2*, higher *AGL19/AGL14/SOC1* expression was observed during early flower development (before stage 9) than during late developmental stages (after stage 12) (Fig. 4l and Supplementary Figs. 7e, 8d), which further revealed possible similar and overlapping functions of *FYF* and *AGL19/AGL14/SOC1*. GUS staining was detected in sepal/petal organs and was absent in the AZ of AGL19::*GUS* (Fig. 4m, n) and SOC1::*GUS* flowers (Supplementary Fig. 8e–g), suggesting that *AGL19/14* and *SOC1* might have part of the functions of *FYF* in regulating flower senescence but not abscission. Although SOC1 and AGL19 might also be involved in regulating flower senescence, they have very low or complete no interactions with AGL6 (Fig. 2n, columns 4, 5) or SEP1 (Fig. 2o, columns 4, 5). This result indicated that the *FYF/FYL1/FYL2* and *AGL19/14/SOC1* groups might have evolved to have their own interacting partners in regulating flower senescence/abscission.

### *FYF* activates *FYL1* and *AGL19/14/SOC1* expression to enhance the regulation of flower abscission and senescence

Since *FYF* has the same function as *FYL1* and *AGL19/14/SOC1* to regulate abscission and senescence of the sepal/petal, respectively, we were also interested in determining how they work together. When the expression pattern of endogenous *FYL1* and *AGL19/14/SOC1* was analyzed in 35 S::*FYF* flowers, we found that the expression of all three genes was clearly upregulated (Fig. 4o). Our results revealed that *FYL1* was activated by *FYF* in the AZ during flower development and could enhance the function of the *FYF* gene in suppressing sepal/petal abscission, whereas *AGL19/14/SOC1* were activated by *FYF* in sepals/petals during flower development, which could enhance the function of the *FYF* gene in suppressing sepal/petal senescence. Interestingly, we found that *FYF*, *FYL1, AGL14,* and *SOC1* expression was also upregulated in 35 S::*AGL19* flowers (Fig. 4p). This result suggested that *FYF* and other *FYF-*like genes with the strong repressor role, such as *AGL19*, could reciprocally activate each other to enhance the suppression of sepal/petal senescence.

### *FYF* activates *FYL2* expression to regulate flower senescence possibly through a feedback loop

Exploring how FYF competes with FYL2 to oppositely regulate sepal/petal senescence is interesting. When the expression pattern of endogenous *FYL2* was analyzed in 35S::*FYF* flowers, we found that *FYL2* expression was upregulated (Fig. 4o). Our results revealed that *FYL2* was activated by *FYF* during flower development and that FYL2 could possibly form a feedback loop to contend with endogenous FYF function and to more appropriately control flower senescence. This assumption was further supported by the downregulation of *FYL2* expression in *fyf/agl15* double mutants (Supplementary Fig. 9a). We also found that *FYL2* expression was upregulated in 35S::*AGL19* flowers (Fig. 4p). This result suggested that *FYF* and *AGL19* might control sepal/petal senescence by regulating FYL2 in a similar way.

## Discussion

The *Arabidopsis* MADS box gene *FYF* can regulate flower organ senescence and abscission^1^. Ectopic expression of *FYF* caused a delay of senescence and a deficiency of abscission in flowers of transgenic *Arabidopsis* and *Eustoma grandiflorum*^1^. This study further showed that two tandem repeat *FYF*-like genes, *FYL1,* and *FYL2*, and three other *FYF*-like genes, *AGL19/14* and *SOC1*, in *Arabidopsis* were also involved in the regulation of flower organ abscission and/or senescence, and their functions were complementary or antagonistic to *FYF*.

**Fig. 5:**
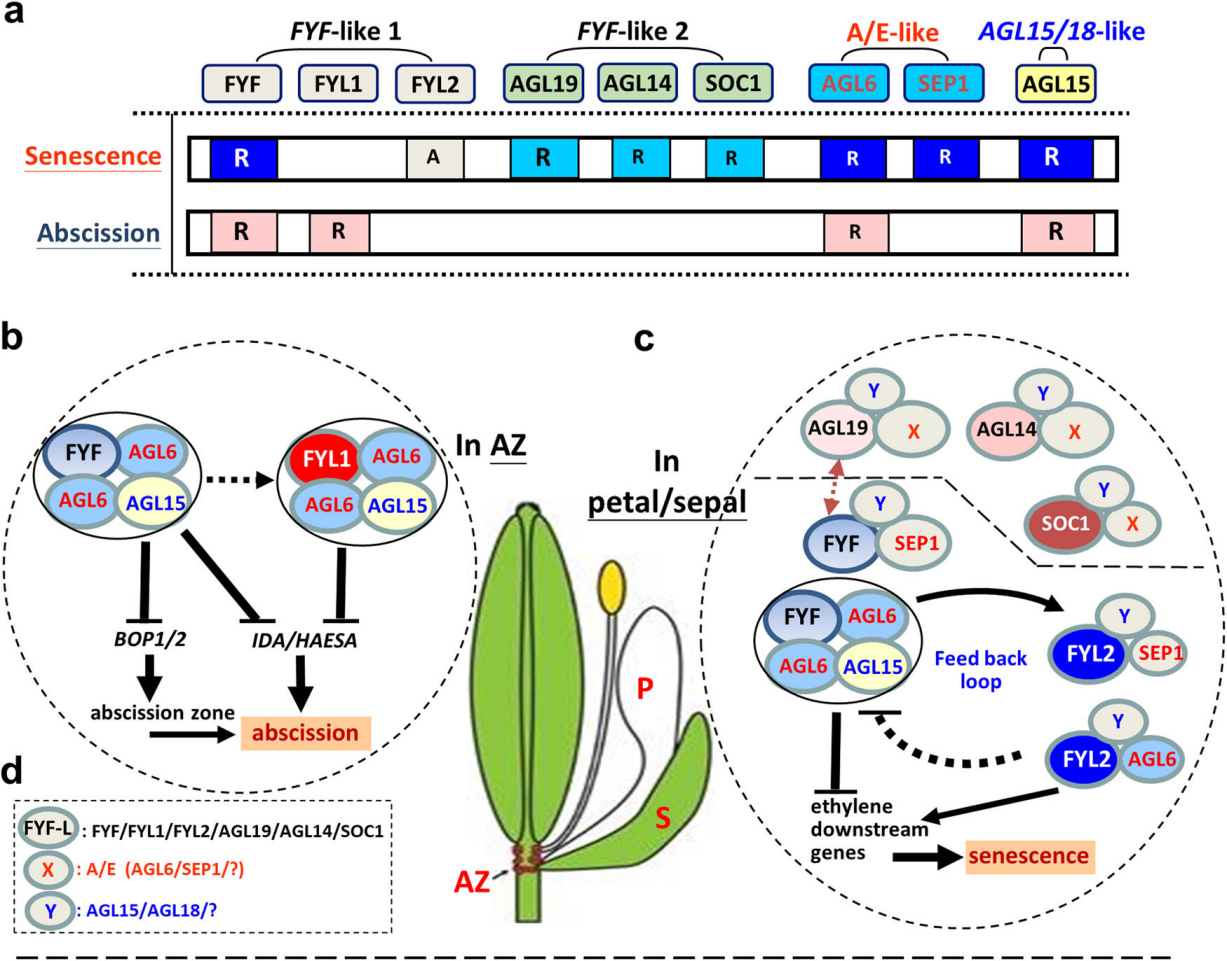
The functional evolution and regulatory network of the *FYF*-like genes in regulating flower senescence/abscission. **a** In *Arabidopsis*, six *FYF*-like genes in two subgroups (*FYF/FYL1/FYL2* and *AGL19/AGL14/SOC1*) were all involved in regulating flower senescence and/or abscission. In the *FYF* subgroup, *FYF* acts as a repressor (R) in suppressing both flower senescence (indicated by a blue box) and abscission (indicated by a pink box), and *FYL1* acts as a repressor (R) and only suppresses flower abscission (indicated by a pink box). *FYL2* functions as an activator (A) in promoting flower senescence (indicated by a gray box). In the *SOC1* subgroup, *AGL19, AGL14,* and *SOC1* function as repressors (R) and have only one function in suppressing flower senescence (indicated by a light blue box), with the effect of *AGL19* being stronger than that of *AGL14/SOC1*. In addition, the A/E functional genes *AGL6/SEP1* and *AGL15/18*-like gene *AGL15* (as a repressor) can regulate senescence (indicated by a blue box) by interacting with FYF/FYL2, whereas *AGL6* and *AGL15* can also regulate abscission (indicated by a pink box) by interacting with FYF/FYL1. The size of the letter R in the box correlated with the strength of the repressor for the MADS box proteins. **b** In the AZ of the perianth, FYF and FYL1 complement each other by forming two identified heterotetrameric abscission complexes, FYF/AGL6/AGL6/AGL15 and FYL1/AGL6/AGL6/AGL15, suppressing flower abscission through the downregulation (⊣) of *BOP1/2* and *IDA/HAESA* expression. **c** In sepals/petals, FYF, AGL19, AGL14, and SOC1 functioned antagonistically to FYL2 in suppressing flower senescence. An identified FYF/AGL6/AGL6/AGL15 together with FYF/SEP1/Y, AGL19/X/Y, AGL14/X/Y, and SOC1/X/Y heterotetrameric senescence complexes suppressed sepal/petal senescence through the downregulation (⊣) of ethylene downstream gene expression. In contrast, FYL2/AGL6/Y and FYL2/SEP1/Y heterotetrameric complexes promoted sepal/petal senescence by the activation (→) of ethylene downstream gene expression, possibly through a negative feedback loop to FYF/X/Y, AGL19/X/Y, AGL14/X/Y, and SOC1/X/Y. **d** In these cases from (**c**), the heterotetrameric complexes are composed of FYF-like, X (in red) and Y (in blue) proteins. FYF-like can be either one of the FYF/FYL1/FYL2/AGL19/AGL14/SOC1, X can be AGL6, SEP1, or any unidentified A/E proteins, whereas Y can be AGL15, AGL18, or any unidentified AGL15/18-like proteins.

*FYL1* was found to act as a repressor to suppress abscission in sepals/petals with a complementary function to *FYF* (Fig. 5a). Unexpectedly, *FYL2* could function as an activator and antagonize *FYF* in promoting the senescence of sepals/petals (Fig. 5a). The functions of *FYL1/2* are correlated with their expression pattern since *FYL1* was specifically expressed in the AZ of sepals/petals, whereas *FYL2* expression was detected in the organs of sepals/petals. The amino acid identity and the phylogenetic tree relationship^6^ revealed that *FYL1/2* were possibly the result of two duplication events. The first event generated an *FYL1/2* ancestor from *FYF*, and the second event produced the two tandem repeats *FYL1* and *FYL2* from this *FYL1/2* ancestor. The conserved role of *FYF/FYL1/FYL2* during evolution in regulating flower senescence and/or abscission was further supported by their ability to interact with the same MADS box proteins AGL6 and SEP1. The original *FYF* gene must have contained both regulatory elements in its promoter or introns^1,33,41–45^, which are required for its expression in the organs and AZ of sepals/petals. In the AZ, FYF specifically interacts with AGL6 to suppress abscission of the sepals/petals (Fig. 5a, b). In the sepal/petal organs, FYF can interact with either AGL6 or SEP1 to suppress senescence (Fig. 5a, c). In the tandem repeat genes *FYL1* and *FYL2*, the subfunctional alteration of the regulatory elements during evolution resulted in restriction of the expression of FYL1 in the AZ and FYL2 in sepal/petal organs. FYL1 should maintain the conserved ability of FYF to interact with AGL6 together to suppress the abscission of sepals/petals (Fig. 5a, b). Conversely, FYL2 only retained the conserved ability of FYF to interact with AGL6 and SEP1 in regulating sepal/petal senescence (Fig. 5a, c). However, FYL2 evolved into a role antagonistic to FYF and possibly helped to control FYF activity through a feedback loop to protect the flower buds from senescence and ensure the final senescence of the mature flowers (Fig. 5a, c).

In addition to *FYL1/2*, three putative *FYF*-like genes in the *SOC1* subgroup, *AGL19, AGL14*, and *SOC1*, which are closely related to *FYF/FYL1/FYL2* genes based on the phylogenetic tree relationship^6,16^, were also characterized. Based on the results of the functional analysis and the expression patterns of these three genes, we found that *AGL19, AGL14,* and *SOC1* were all involved in the regulation of senescence but not abscission of flower organs (Fig. 5a, c). AGL19 might play a stronger repressive role than AGL14/SOC1, which functions similarly to FYF in suppressing flower senescence (Fig. 5a). Although AGL19/14 and SOC1 were found to be involved in regulating flower senescence, similar to FYF/FYL2, they seemed to perform their function in a different way in terms of finding interacting partners to form functional complexes. For example, FYF/FYL1/FYL2 can interact sufficiently with AGL6/SEP1, whereas AGL19/14/SOC1 can not interact with AGL6/SEP1. This finding indicated that the AGL19/14/SOC1 subgroup might have their own interacting partners in regulating flower senescence that differ from those of FYF/FYL1/FYL2 during evolution.

One interesting finding is that these *FYF*-like genes could regulate the expression of each other. For example, *FYF* could positively regulate the expression of *FYL1* and *AGL19/14/SOC1* to enhance suppression of the abscission and senescence of flower organs, respectively, whereas *AGL19* could positively regulate the expression of *FYF* and *AGL14/SOC1* to enhance the suppression of flower organ senescence. This positive reciprocal regulatory network among the *FYF*-like genes should provide a mechanism to ensure the suppression of senescence/abscission during the early stage of flower organ development (Fig. 5b, c). In addition, we also found a possible feedback loop regulatory mechanism between FYF and its opposite functional activator FYL2. FYF could activate the expression of *FYL2*, which possibly sequentially antagonized the activity of FYF. In this case, FYF activity will be countervailed at an appropriate level by FYL2, which is high in early and low in late flower development and ensures that sepal/petal senescence will occur after flower maturation and will not occur in the flower bud stage (Fig. 5c).

In addition to the *FYF*-like genes studied, we also found that one other MADS box protein, AGL15, which has also been characterized to be able to regulate flower organ senescence/abscission (Fig. 5a)^38,39^, was able to interact with AGL6 to form stable heterotetrameric abscission complexes (FYF-AGL6-AGL6-AGL15 and FYL1-AGL6-AGL6-AGL15) and a senescence complex (FYF-AGL6-AGL6-AGL15) with FYF/FYL1 (Fig. 5b, c). Interestingly, AGL18, a closely related gene to AGL15^38,39^, has no interaction with AGL6 to form further heterotetrameric abscission/senescence complexes with FYF/FYL1. Thus, despite redundant functions, AGL15 and AGL18 may interact with their specific partners to form different protein complexes to regulate flower organ abscission/senescence (Fig. 5d).

A scenario was proposed to elucidate the evolutionary modification of the functions and complicated network of *FYF*-like genes in regulating flower senescence/abscission. In *Arabidopsis*, an *FYF*-like ancestor duplicated into two subgroups of genes (*FYF/FYL1/FYL2* and *AGL19/AGL14/SOC1*) and eventually evolved to divergent functions through subfunctionalization in regulating various flower development processes, including senescence/abscission. In the *FYF* subgroup, *FYF* has both functions in suppressing flower senescence and abscission, whereas *FYL1* only suppresses flower abscission, and *FYL2*’s function is converted into the activation of flower senescence (Fig. 5a). In the *SOC1* subgroup, *AGL19, AGL14,* and *SOC1* showed only one function in suppressing flower senescence, with the effect of *AGL19* being stronger than that of *AGL14/SOC1* (Fig. 5a). FYF-like proteins can form heterotetrameric complexes with A/E functional proteins (AGL6, SEP1, and unidentified X) and AGL15/18-like proteins (AGL15/18 and unidentified Y) to perform their functions (Supplementary Fig. 2 and Fig. 5d). In the AZ, FYF/AGL6/AGL6/AGL15 and FYL1/AGL6/AGL6/AGL15 were two identified heterotetrameric abscission complexes that suppressed flower abscission (Fig. 5b). In sepals/petals, we identified FYF/AGL6/AGL6/AGL15 together with FYF/SEP1/Y, AGL19/X/Y, AGL14/X/Y, and SOC1/X/Y heterotetrameric senescence complexes in suppressing flower senescence (Fig. 5c).

Our findings reveal the potential immense complexity of the different combinations of FYF-like, A/E functional, and AGL15/18-like proteins in forming heterotetrameric abscission/senescence complexes (Fig. 5d). This complicated gene redundancy might explain why it is difficult to identify the senescence/abscission mutant phenotype in a single gene mutation for these genes. In an attempt to mutate *FYF* (key gene in *FYF*-like) and *AGL15* (key gene in *AGL15/18*-like) simultaneously, T-DNA mutants for each gene were crossed to generate *fyf/agl15* double mutations. Very interestingly, early senescence and abscission of the flowers was observed in these *fyf/agl15* double mutants (Supplementary Fig. 9). This result strongly supported our assumption that abscission/senescence heterotetrameric complexes are at least composed of different combinations of FYF-like and AGL15/18-like proteins. Simultaneous mutations in FYF and AGL15 proteins will disrupt the functions of various combinations of the complexes and result in early senescence/abscission mutant phenotypes. In conclusion, our findings not only greatly expand the current knowledge concerning the multifunctional evolution of *FYF*-like genes in regulating flower senescence/abscission but also provide an excellent example for the study of diverse functionalizations of duplicate gene pairs in plants.

## Methods

### Plant materials and growth conditions

The T-DNA insertion mutants of *FYF* (*fyf*, SALK_047915) and *AGL15* (*agl15*, SALK_076234C) mutants *Arabidopsis* seeds were obtained from the Arabidopsis Biological Resource Center, Ohio State University, Columbus, OH, USA. Seeds for *Arabidopsis* were germinated and grown as described previously^1,2,46^. *Arabidopsis* seeds were sterilized and placed on agar plates containing 1/2 X Murashige & Skoog medium^47^ at 4 °C for 2 days. Before being transplanted to soil, the seedlings were grown in growth chambers under long-day conditions (16 h light/8 h dark) at 22 °C for 10 days. The light intensity of the growth chambers was 150 μE m^−2^ s^−1^.

### Cloning of the cDNA for *FYL1*, *FYL2*, *AGL6*, *AGL19*, *AGL14*, *SOC1*, and *AGL15* from *Arabidopsis*

For 35S::*MADS* constructs, the cDNAs for *FYL1, FYL2, AGL6, AGL19, AGL14, SOC1,* and *AGL15* were obtained by PCR amplification using gene-specific 5′ and 3′ primers. The primers contained the *Xba*I and *Kpn*I recognition sites to facilitate the cloning of the cDNAs. The *Xba*I-*Kpn*I fragment containing the cDNA was cloned into the binary vector pEpyon-22K^1^ under the control of the CaMV 35S promoter and used for plant transformation. Sequences for the primers are listed in the Supplementary Table 1.

### Cloning of the promoter DNA fragment from *Arabidopsis*

For the FYL1::*GUS* and FYL2::*GUS* constructs, the promoter regions which included the 5′UTR and first intron for *FYL1* (2.65 kb) and *FYL2* (2.43 kb) were obtained by PCR amplification using specific primer pairs from the genomic DNA followed by cloning into the pGEM-T easy vector (Promega, Madison, WI, USA). These promoter fragments were then subcloned into the linker region before the β-Glucuronidase (GUS) coding region in the binary vector pEpyon01k^1,2^. For the AGL6::*GUS*, AGL15::*GUS*, AGL19::*GUS,* and SOC1::*GUS* constructs, the promoter regions which included the 5’UTR and first intron for *AGL6* (4.96 kb), *AGL15* (0.94 kb), *AGL19* (4.59 kb) and *SOC1* (5.68 kb) were obtained by PCR amplification and these promoter fragments were subcloned into the linker region before the β-Glucuronidase (GUS) coding region in the binary vector pEpyon01k in the same manner as FYL1/2::*GUS*. Sequences for the primers are listed in Supplementary Table 1. For the IDA::*FYL1* construct, the *IDA* promoter (1.43 kb) was obtained by PCR amplification as described previously^1^. The cDNA for *FYL1* was obtained by PCR amplification. The *IDA* promoter and the cDNA for *FYL1* were subcloned into the modified binary vector pEpyon-12K^1^. Sequences for the primers are listed in Supplementary Table 1.

### Construction of the *MADS* + *SRDX* constructs

For the 35S::*FYL1*+*SRDX*, 35S::*FYL2*+*SRDX*, 35S::*AGL6*+*SRDX*, 35S::*AGL15*+*SRDX*, 35S::*AGL19*+*SRDX*, 35S::*AGL14*+*SRDX*, 35S::*SOC1*+*SRDX* constructs, the cDNAs for *FYL1/FYL2/AGL6/AGL15/AGL19/AGL14/SOC1* were obtained by PCR amplification and cloned into the pEpyon-2aK plasmid upstream of the SRDX (LDLDLELRLGFA*) sequence, under the control of the CaMV 35S promoter as described previously^1,2^. The sequences for the primers are listed in Supplementary Table 1.

### Construction of the *MADS* + *VP16* constructs

For the 35S::*FYL1*+*VP16*, and 35S::*FYL2*+*VP16* construct, the cDNAs for *FYL1/FYL2* were obtained by PCR amplification and cloned into the pEpyon-2bK plasmid upstream of the VP16-AD fragment sequence, under the control of the CaMV 35S promoter as described previously^1,2^. The sequences for the primers were listed in Supplementary Table 1.

### Plant transformation and transgenic plant analysis

A floral dip method as described elsewhere^48^ was used to introduce constructs made in this study in the *Agrobacterium tumefaciens* strain GV3101 into *Arabidopsis* plants. PCR and RT-PCR analyses were used to verified the transformants that survived in medium containing kanamycin (50 µg/ml). To generate 35S::*FYF*/35S::*FYL2 Arabidopsis*, constructs of 35S::*FYL2* which contained hygromycin resistant gene were co-transformed with 35S::*FYF* (kanamycin resistant) into *Arabidopsis* plants. Transformants that survived in medium containing both kanamycin (50 µg/ml) and hygromycin (5 µg/ml) were selected for further analysis. To generate *fyf/agl15* double mutant *Arabidopsis*, homozygous *fyf* were crossed with the *agl15* T-DNA mutants in the Columbia background and F_1_ plants were used to further generate the F_2_ generation. One quarter of the F_2_ plants were *fyf/agl15* and were further verified and selected for further analysis.

### Histochemical GUS assay

Histochemical staining was performed under standard method described previously^2,49,50^. Samples were incubated in solution (0.05 mM Potassium ferricyanide, 0.05 mM Potassium ferrocyanide, 100 mM Phosphate buffer, pH 7.0) containing 2 mM X-Gluc (5-bromo-4-chloro-3-indolyl ß-D-glucuronic acid) for several hours at 37 °C. The sample was examined under a dissecting microscope.

### Real-time PCR analysis

For real-time quantitative RT-PCR, the reaction was performed on a MJ Opticon system (MJ Research, Waltham, MA) using SYBR^®^ Green Real-time PCR Master Mix (TOYOBO Co., LTD.). The amplification condition was 95 °C for 10 min, followed by 40 cycles of amplification (95 °C for 15 s, 58 °C for 15 s, 72 °C for 30 s and then plate reading) and melted (50–95 °C with plate readings every 1 °C) as described previously^1,2^. Sequences for the primers used for real-time quantitative RT-PCR for *FYF*, *FYL1, FYL2, AGL6, AGL15, AGL19, AGL14, SOC1*, *EDF1*, *EDF2*, *EDF3*, *EDF4*, *ERF1, SAG12, BOP1, BOP2*, *IDA,* and *HAESA*, were listed in Supplementary Table 1. The housekeeping gene *UBQ10* was used as normalization control with the following primers: RT-UBQ10-1 and RT-UBQ10-2^51^. Data were analyzed using Gene Expression Macro software (version 1.1, Bio-Rad).

### Ethylene responses

As described previously^1,52^, wild-type and transgenic *Arabidopsis* plants were sealed in plastic chambers and gassed with air or air containing 10 ppm ethylene for 3 days in a 16 h light/8 h dark cycle and phenotype analyzed.

### FRET analysis

The procedure used to prepare FRET-associated fusion constructs was described in previous studies^36,53^. To fuse FYF/FYL1/FYL2/AGL6/SEP1/AGL15/AGL18/AGL19/SOC1 with CFP or YFP, the cDNAs for *Arabidopsis FYF/FYL1/FYL2/AGL6/SEP1/AGL15/AGL18/AGL19/SOC1* were obtained by PCR amplification using gene-specific primers and cloned into the pEpyon-36K and pEpyon-37K vectors upstream of the CFP or YFP sequence under the control of the CaMV 35S promoter. The following gene-specific primers were used: *FYF*, FYF-FRET-F-PstI (5′-CTGCAGATGGTTAGAGGAAAGATAGAGATGAAG-3′) and FYF-FRET-R-ns-SalI (5′-GTCGACGCAGTTTCTATTTGGCAAACCG-3′); *FYL1*, FYL1-FRET-F-XbaI (5′-TCTAGAATGGTGAGAGGGAAGATCGAGATC-3′) and FYL1-FRET-R-ns-kpnI (5′-GGTACCTAGCCGAGTCACGGGCAATC-3′); *FYL2*, FYL2-FRET-F-XbaI (5′-TCTAGACTGCAGATGGGAAGGGGAAGAGTTGA-3′) and FYL2-FRET-R-ns-kpnI (5′-GGTACCTGGTCGGTTCTTCAGAAATCC-3′); *AGL6*, AGL6-FRET-F-XbaI (5′-TCTAGAATGGGAAGAGGGAGAGTGG-3′) and AGL6-FRET-R-ns-kpnI (5′-GGTACCAAGAACCCAACCTTGGACG-3′); *SEP1*, SEP1-FRET-F-pstI (5′-CTGCAGATGGGAAGAGGAAGAGTAGAGCTGAAGAG-3′) and SEP1-FRET-R-ns-SalI (5′-GTCGACGAGCATCCACCCCGGGATGT-3′); *AGL15*, AtAGL15-FRET-F-XbaI (5′-TCTAGAATGGGTCGTGGAAAAATCGAG-3′) and AtAGL15-FRET-R-ns-kpnI (5′-GGTACCAACAGAGAACCTTTGTCTTTTGGC-3′); *AGL18*, AtAGL18-FRET-F-XbaI (5′-TCTAGAATGGGTCGGGGAAAGATAGA-3′) and AtAGL18-FRET-R-ns-kpnI (5′-GGATCCATCAGAAGCCACTTGACTCC-3′); *AGL19*, AtAGL19-FRET-F-XbaI (5′-TCTAGAATGGTGAGGGGCAAAACG-3′) and AtAGL19-FRET-R-ns-kpnI (5′-GGTACCATTTTGAGGAGGGAATTTTTTGGATTGTC-3′); *SOC1*, AtSOC1-FRET-F-XbaI (5′-TCTAGAATGGTGAGGGGCAAAACTC-3′) and AtSOC1-FRET-R-ns-kpnI (5′-GGTACCCTTTCTTGAAGAACAAGGTAACCCAATG-3′). These constructs were transformed into the *Agrobacterium* strain C58C1. Different ectopic proteins were expressed in tobacco cells and a confocal microscope was used to detect the fluorescence signals in the nucleus. To perform the subcellular localization assay, *Agrobacterium*-infiltrated *N. benthamiana* leaves were vacuum infiltrated in 10 mM MgCl2 at room temperature until immersed. As previously described^36,52^, an Olympus FV1000 confocal microscope (Olympus FV1000, Tokyo, Japan) and the FV-ASW 3.0 software were used to visualize fluorophores and to calculate the raw FRET and FRET efficiency values. To evaluate the variation in protein interaction distances among different protein complexes (*n* > 4), the mean value of FRET efficiency in the nucleus was calculated.

### Statistics and reproducibility

Data in the analysis of gene expression in various transgenic plants were analyzed using the two-sided Student’s *t*-test and represented as the mean ± SD. In these cases, *n* = 3 biologically independent samples. The letter “a”, “b” and “c” indicates significant difference from the wild-type (WT) value (a: *P* < 0.05, b: *P* < 0.01, and c: *P* < 0.001).

### Reporting summary

Further information on research design is available in the Nature Research Reporting Summary linked to this article.

## Supplementary information

Supplementary Information

Description of Additional Supplementary Files

Supplementary Data 1

Reporting-summary

## Data Availability

The data supporting the findings of this work are available within the paper and the Supplementary Information files. The data sets generated and analyzed during this study are available from the corresponding author upon request.
