# Supplementary material for: Regulatory network for FOREVER YOUNG FLOWER-like genes in regulating Arabidopsis flower senescence and abscission
Source: Commun Biol. 2022 Jul 5;5:662. doi: 10.1038/s42003-022-03629-w (PMC9256709; doi:10.1038/s42003-022-03629-w)
Supplement: Supplementary file 1 — Supplementary Information [file 42003_2022_3629_MOESM1_ESM.pdf]

Supplementary Fig. 1

```
                    10        20        30        40        50        60
      ....|....|....|....|....|....|....|....|....|....|....|....|
FYF   MVRGKIEMKKIENATSRQVTFSKRRNGLLKKAYELSVLCDAQLSLIIFSQRGRLYEFSSS
FYL1  MVRGKIEIKKIENVTSRQVTFSKRRSGLFKKAHELSVLCDAQVAAIVFSQSGRLHEYSSS
FYL2  MVRGKIEIKKIENVTSRQVTFSKRRSGLFKKAHELSVLCDAQVAAMIFSQKGRLYEFASS
AGL19 MVRGKTEMKRIENATSRQVTFSKRRNGLLKKAFELSVLCDAEVALVIFSPRSKLYEFSSS
AGL14 MVRGKTEMKRIENATSRQVTFSKRRNGLLKKAFELSVLCDAEVALIIFSPRGKLYEFSSS
SOC1  MVRGKTQMKRIENATSRQVTFSKRRNGLLKKAFELSVLCDAEVSLIIFSPKGKLYEFASS

                    70        80        90       100       110       120
      ....|....|....|....|....|....|....|....|....|....|....|....|
FYF   D-MQKTIERYRKYTKDH-ETSNHDSQIHLQQLKQEASHMITKIELLEFHKRKLLGQGIAS
FYL1  Q-MEKIIDRYGKFSNAFYVAERPQVERYLQELKMEIDRMVKKIDLLEVHHRKLLGQGLDS
FYL2  D-IRNTIKRYAEYKREYFVAETHPIEQYVQGLKKEMVTMVKKIEVLEVHNRKMMGQSLDS
AGL19 S-IAATIERYQRRIKE--IGNNHKRNDNSQQARDETSGLTKKIEQLEISKRKLLGEGIDA
AGL14 SSIPKTVERYQKRIQD--LGSNHKRNDNSQQSKDETYGLARKIEHLEISTRKMMGEGLDA
SOC1  N-MQDTIDRYLRHTKDR-VSTKPVSEENMQHLKYEAANMMKKIEQLEASKRKLLGEGIGT

                   130       140       150       160       170       180
      ....|....|....|....|....|....|....|....|....|....|....|....|
FYF   CSLEELQEIDSQLQRSLGKVRERKAQLFKEQLEKLKAKEKQLLEENVKLH-------QKN
FYL1  CSVTELQEIDTQIEKSLRIVRSRKAELYADQLKKLKEKERELLNERKRLLEEQNRERLMR
FYL2  CSVKELSEIATQIEKSLHMVRLRKAKLYEDELQKLKAKERELKDERVRLS-------LKK
AGL19 CSIEELQQLENQLDRSLSRIRAKKYQLLREEIEKLKAEERNLVKENKDLK-------EKW
AGL14 SSIEELQQLENQLDRSLMKIRAKKYQLLREETEKLKEKERNLIAENKMLM-------EKC
SOC1  CSIEELQQIEQQLEKSVKCIRARKTQVFKEQIEQLKQKEKALAAENEKLS-------EKW

                   190       200       210       220       230
      ....|....|....|....|....|....|....|....|....|....|
FYF   VIN---PWRGSSTDQQQEKYKVIDLNLEVETDLFIGLPNRNC--------
FYL1  PVVPATLQICDK--GNTEGGHRTKHSSEVETDLFIGLPVTRL--------
FYL2  TIYTHLCQVGERPMGMPSG---SKEKEDVETDLFIGFLKNRP--------
AGL19 LGMGTATIASSQSTLSSSEVNIDDN-MEVETGLFIGPPETRQSKKFPPQN
AGL14 EMQGRGIIGRISSSSSTSELDIDDNEMEVVTDLFIGPPETRHFKKFPPSN
SOC1  GSHESEVWSNKNQESTGRGDEESSPSSEVETQLFIGLPCS--SRK-----
```

Supplementary Fig. 1 Amino acid sequences alignment of six *Arabidopsis* FYF-like proteins.

Alignment of the amino acid sequences for *Arabidopsis* FYF (AGL42), FYL1 (AGL71), FYL2 (AGL72), AGL19, AGL14 (XAL2) and SOC1 (AGL20) proteins. The conserved MADS box domain was underlined in red. The dark highlights indicate identical residues and gray highlights indicate similar residues. Dashes were introduced to improve alignment. The amino acid sequences were aligned by the BioEdit program using ClustalW Multiple Alignment.

Supplementary Fig. 2

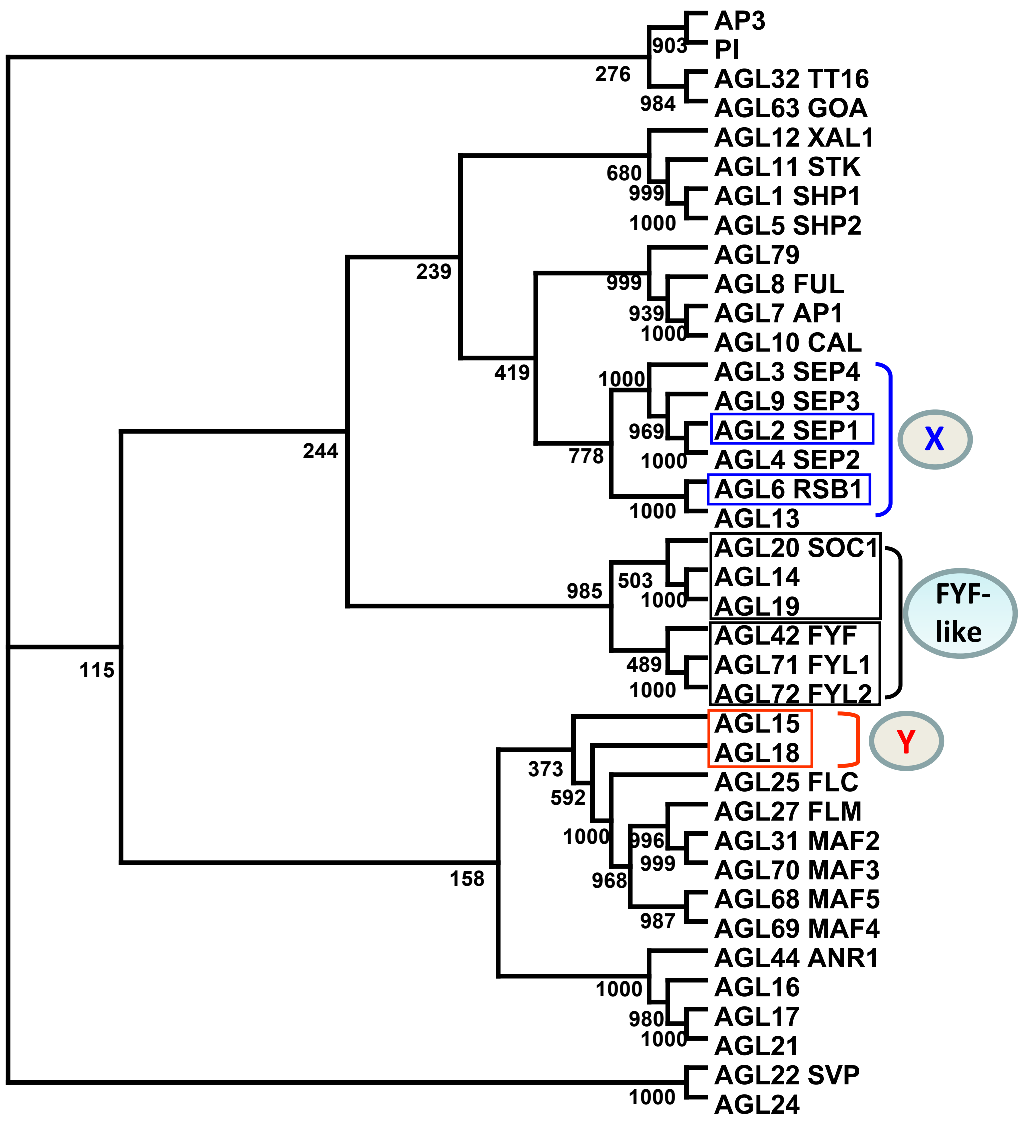

Supplementary Fig. 2 Phylogenetic analysis of *Arabidopsis* FYF-like proteins and their interacting A/E and AGL15/18-like proteins in heterotetrameric senescence/abscission complexes.

Amino acid sequences of MICK group of the *Arabidopsis* MADS box proteins were obtained via the National Center for Biotechnology Information server (http://www.ncbi.nlm.nih.gov/). The FYF-like proteins (in black boxes) were divided into two subgroups, FYF subgroup, including FYF (AGL42), FYL1 (AGL71), FYL2 (AGL72) and SOC1 subgroup, including SOC1 (AGL20), AGL19, AGL14 (XAL2). The A/E proteins (in blue boxes) interacted with FYF-like proteins were indicated as "X" which could be AGL6, AGL13, SEP1-4 or any other closely related proteins. The AGL15/18-like proteins (in red box) interacted with FYF-like proteins were indicated as "Y" which could be AGL15, AGL18 or any other closely related proteins. The multiple sequence alignment was performed by using ClustalW of DNA Data Bank of Japan (http://clustalw.ddbj.nig.ac.jp/top-e.html). The phylogenetic graph was generated by using the TREEVIEW program. Numbers on major branches indicate bootstrap percentages for 1,000 replicate analyses.

Supplementary Fig. 3

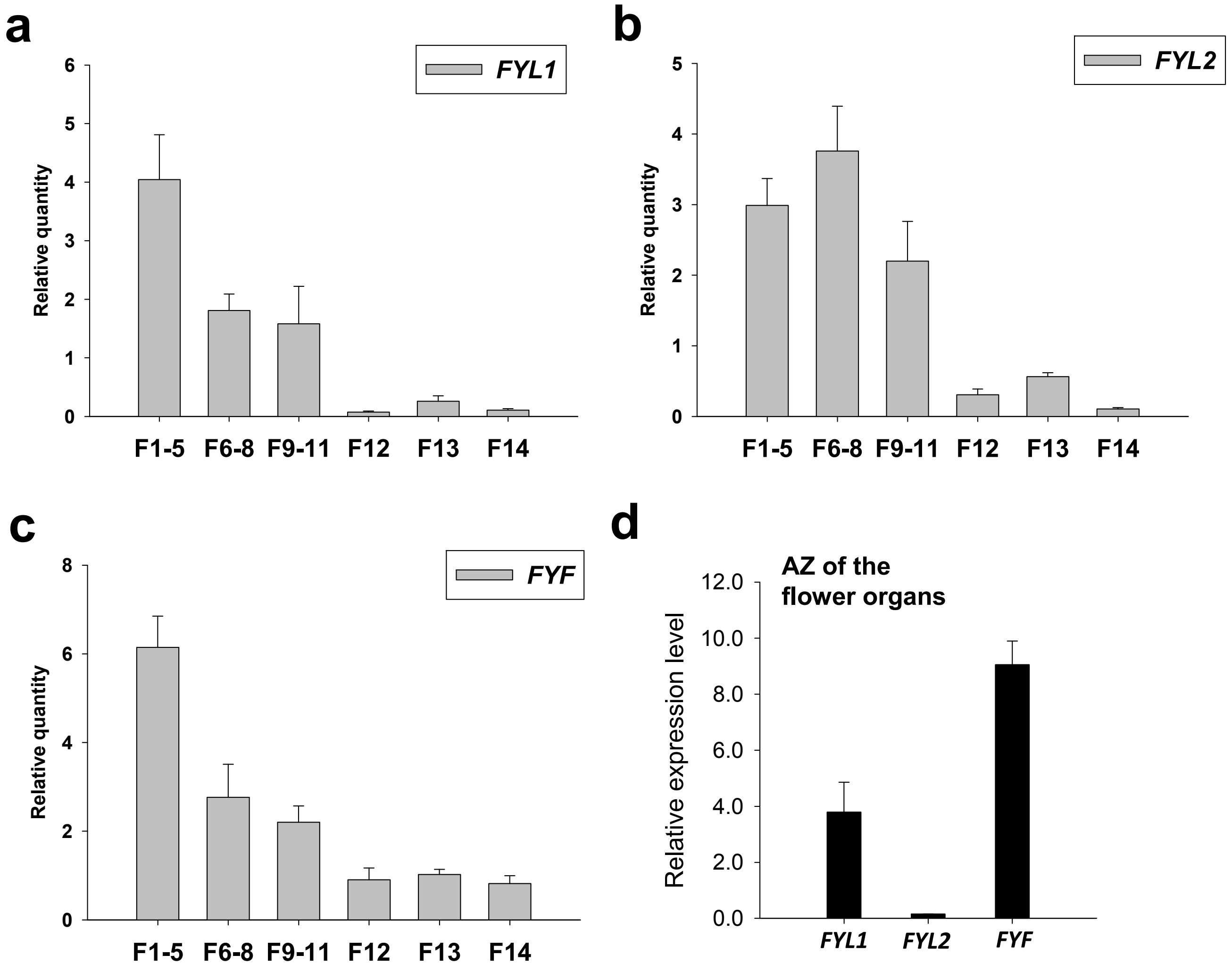

Supplementary Fig. 3 The expression patterns for *FYF, FYL1* and *FYL2* during flower development.

a-c The detection of *FYL1* (**a**)*, FYL2* (**b**) and *FYF* (**c**) expression in wild-type flowers at six different developmental stages (1-5, 6-8, 9-11, 12, 13 and 14). The mRNA levels were determined by real-time quantitative PCR.

d The detection of *FYL1, FYL2* and *FYF* expression in the abscission zone (AZ) of wild-type flower organs. The mRNA levels were determined by real-time quantitative PCR.

Supplementary Fig. 4

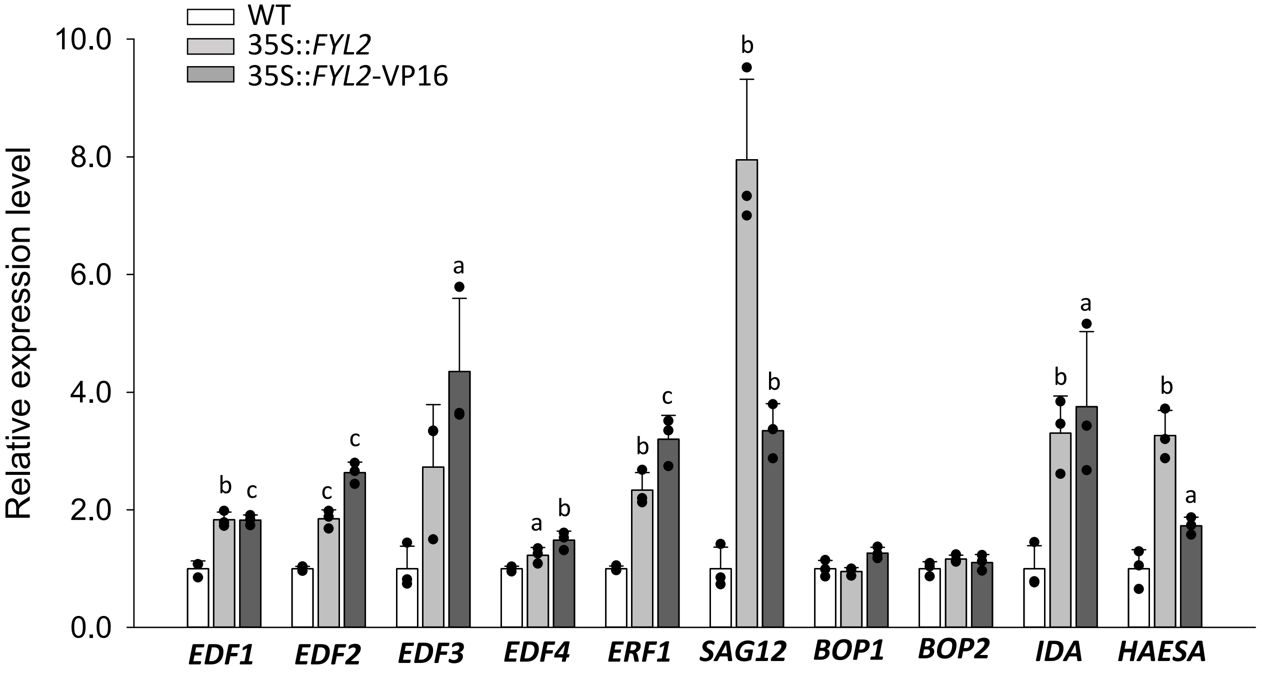

Supplementary Fig. 4 Detection of senescence and abscission genes expression in 35S::*FYL2* and 35S::*FYL2-DR+VP16 Arabidopsis*.

Detection of *EDF1-4, ERF1* and *SAG12* and *BOP1/2, IDA* and *HAESA* expression in 35S::*FYL2* and 35S::*FYL2-DR+VP16 Arabidopsis.* Error bars show ± SD. n=3 biologically independent samples. The expression of each gene in the transgenic plants is given relative to that of the wild-type plant, which was set at 1. The letter “a”, “b” and “c” indicates significant difference from the wild-type (WT) value (a: $P<0.05$, b: $P<0.01$ and c: $P<0.001$). The two-sided Student's *t*-test was used.

Supplementary Fig. 5

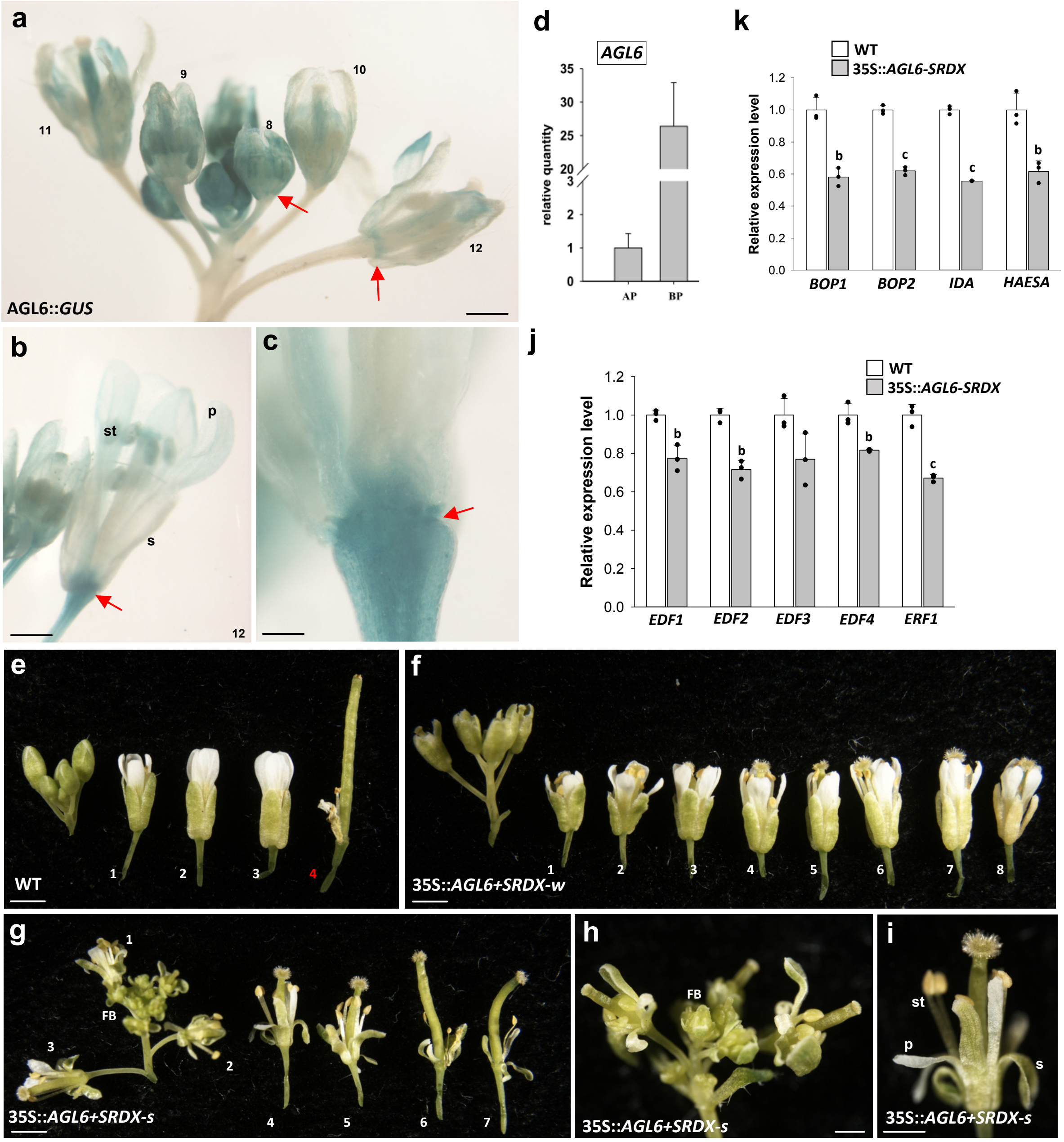

Supplementary Fig. 5 Characterization of the *AGL6* gene through transgenic plants and gene expression analysis in *Arabidopsis*.

a GUS was strongly stained in organs and AZ (arrowed) of sepal/petal in flower buds before stage 9 of AGL6::*GUS Arabidopsis*. GUS staining gradually decreased in the mature flowers (after stage 10) during the late stage of flower development. The numbers indicate the different developmental stages of *Arabidopsis* flowers. Bar = 0.5 mm.

b-c Magnified view of flower (**b**) and the AZ (**c**) (arrowed) in a stage 12 AGL6::*GUS* flower. GUS was strongly stained in the AZ (arrowed) and relatively weak stained in flower organs. s: sepal, p: petal, st: stamen. Bar = 0.5 mm. in **(b)**, 0.1 mm in **(c)**.

d Detection of *AGL6* expression before (BP) and after (AF) pollination.

e-g Flowers along the inflorescence of the wild-type (**e**), 35S::*AGL6+SRDX-w* which showed weak phenotype (**f**) and 35S::*AGL6+SRDX-s* which showed strong phenotype (**g**) plants. The numbers indicate the positions of the flowers. Bars = 1 mm.

h-i Magnified view of the inflorescence with floral buds (FB) (**h**) and a mature flower (**i**) of 35S::*AGL6+SRDX-s* plant from (**g**). s: sepal, p: petal, st: stamen. Bars = 0.5 mm.

j-k Detection of *EDF1-4* and *ERF1* (**j**), *BOP1/2*, *IDA* and *HAESA* (**k**) expression in 35S::*AGL6+SRDX Arabidopsis.* Error bars show ± SD. n=3 biologically independent samples. The expression of each gene in the transgenic plants is given relative to that of the wild-type plant, which was set at 1. The letter "b" and "c" indicates significant difference from the wild-type (WT) value (b: P<0.01 and c: P<0.001). The two-sided Student's *t*-test was used.

Supplementary Fig. 6

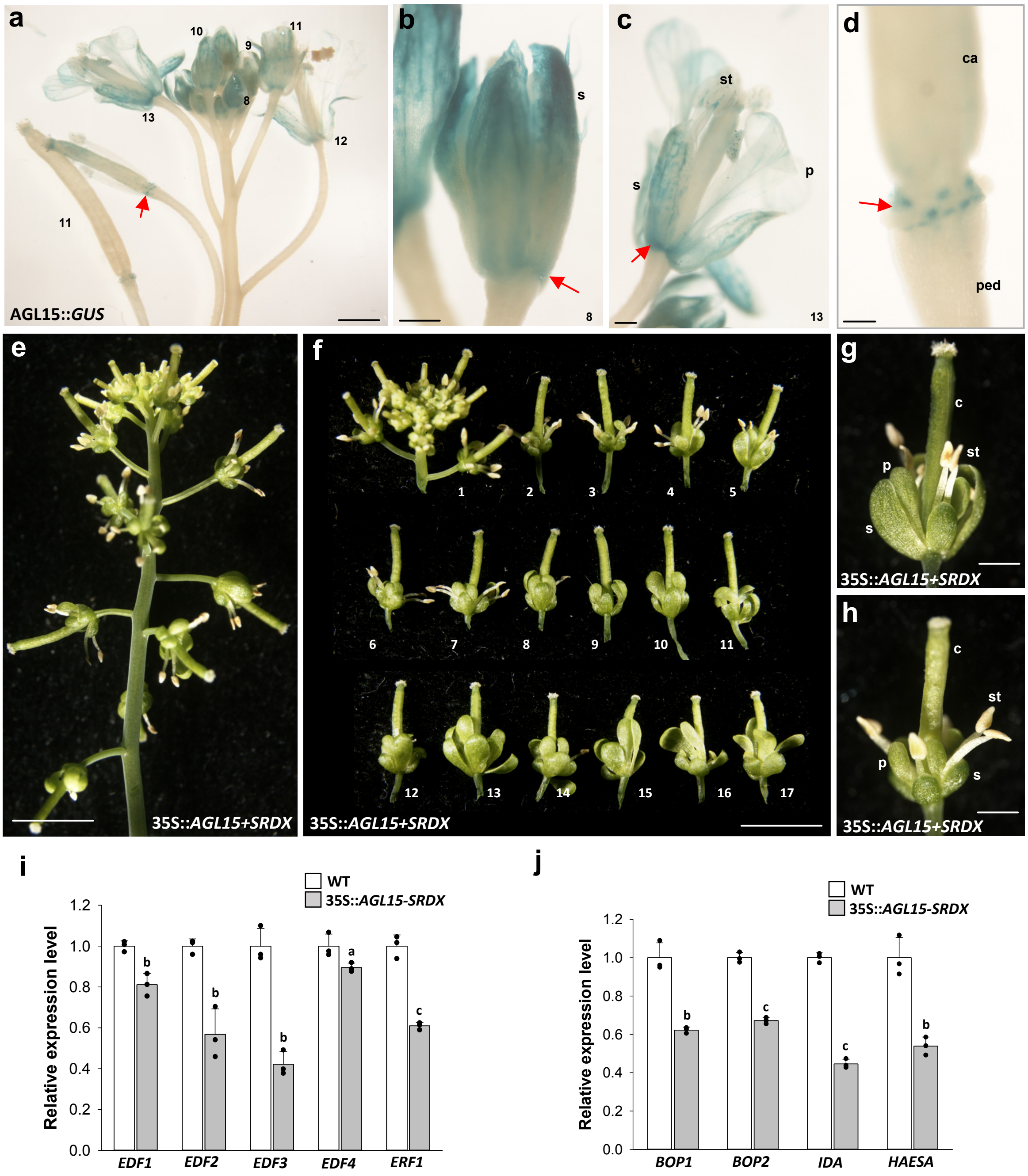

Supplementary Fig. 6 Characterization of the *AGL15* gene through transgenic plants and gene expression analysis in *Arabidopsis*.

a GUS was strongly stained in organs and AZ (arrowed) of sepal/petal in flower buds before stage 10 of AGL15::*GUS Arabidopsis*. GUS staining gradually decreased in the mature flowers (after stage 11) during the late stage of flower development. The numbers indicate the different developmental stages of *Arabidopsis* flowers. Bars = 1 mm.

b-c Magnified view of stage 8 (**b**) and 13 (**c**) AGL15::*GUS* flowers. GUS was stained in organs and AZ (arrowed) of these flowers. s: sepal, p: petal, st: stamen. Bars = 0.2 mm.

d Magnified view of the GUS staining in AZ (arrowed) of a AGL15::*GUS* flower. ca: carpel, ped: pedicel. Bars = 0.1 mm.

e-f The inflorescence with the flowers (**e**) and flowers along the inflorescences (**f**) of the 35S::*AGL15+SRDX* plants. The numbers indicate the positions of the flowers. Bars = 2.5 mm.

g-h Magnified view of delayed senescent/abscised flowers of the 35S::*AGL15+SRDX* plants. s: sepal, p: petal, st: stamen, c: carpel. Bars = 0.5 mm.

i-j Detection of *EDF1-4* and *ERF1* (**i**), *BOP1/2*, *IDA* and *HAESA* (**j**) expression in 35S::*AGL15+SRDX Arabidopsis.* Error bars show ± SD. n=3 biologically independent samples. The expression of each gene in the transgenic plants is given relative to that of the wild-type plant, which was set at 1. The letter "a", "b" and "c" indicates significant difference from the wild-type (WT) value (a: $P<0.05$, b: $P<0.01$ and c: $P<0.001$). The two-sided Student's *t*-test was used.

Supplementary Fig. 7

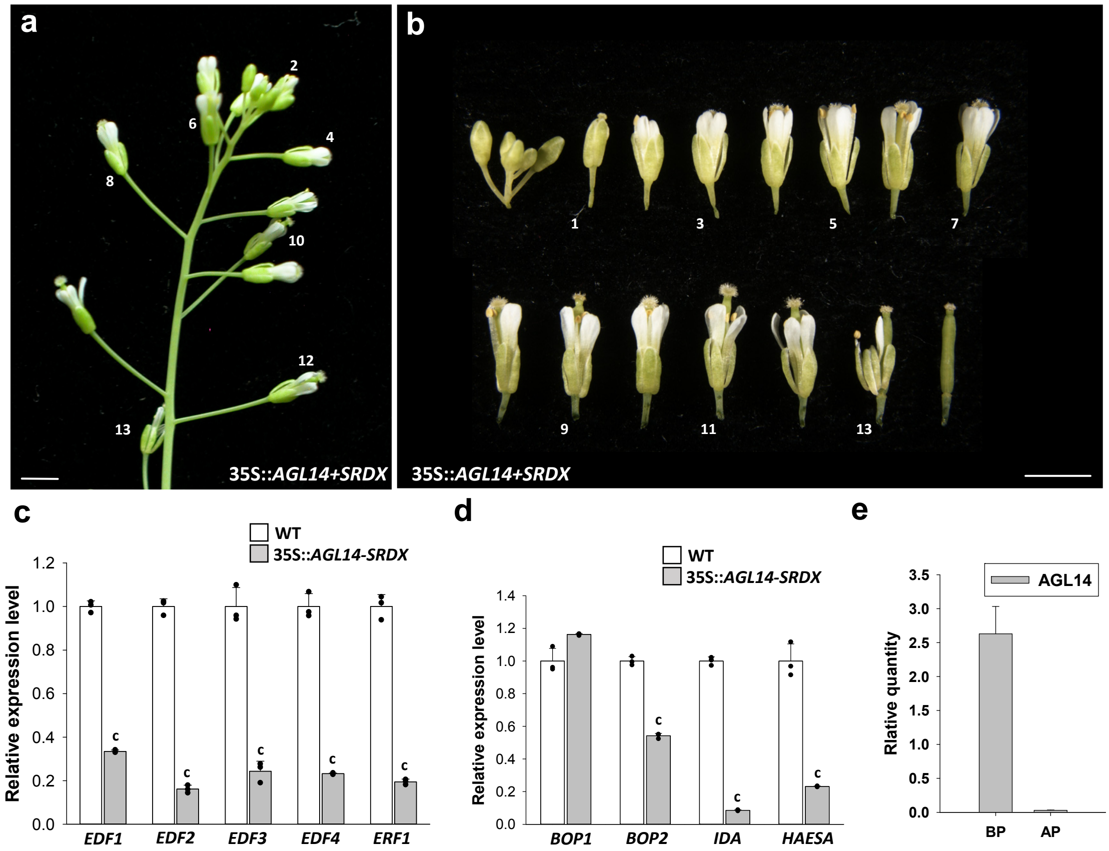

Supplementary Fig. 7 Characterization of the *AGL14* gene through transgenic plants and gene expression analysis in *Arabidopsis*.

a-b The inflorescence with the flowers (a) and the flowers along the inflorescences (b) of 35S::*AGL14+SRDX* plants. The numbers indicate the positions of the flowers. Bars = 2 mm.

c-d The detection of *EDF1-4* and *ERF1* (c), *BOP1/2*, *IDA* and *HAESA* (d) expression in 35S::*AGL14+SRDX Arabidopsis.* Error bars show ± SD. n=3 biologically independent samples. The expression of each gene in the transgenic plants is given relative to that of the wild-type plant, which was set at 1. The letter “c” indicates significant difference from the wild-type (WT) value (c: $P<0.001$). The two-sided Student's *t*-test was used.

e The detection of *AGL14* expression before (BP) and after (AF) pollination.

Supplementary Fig. 8

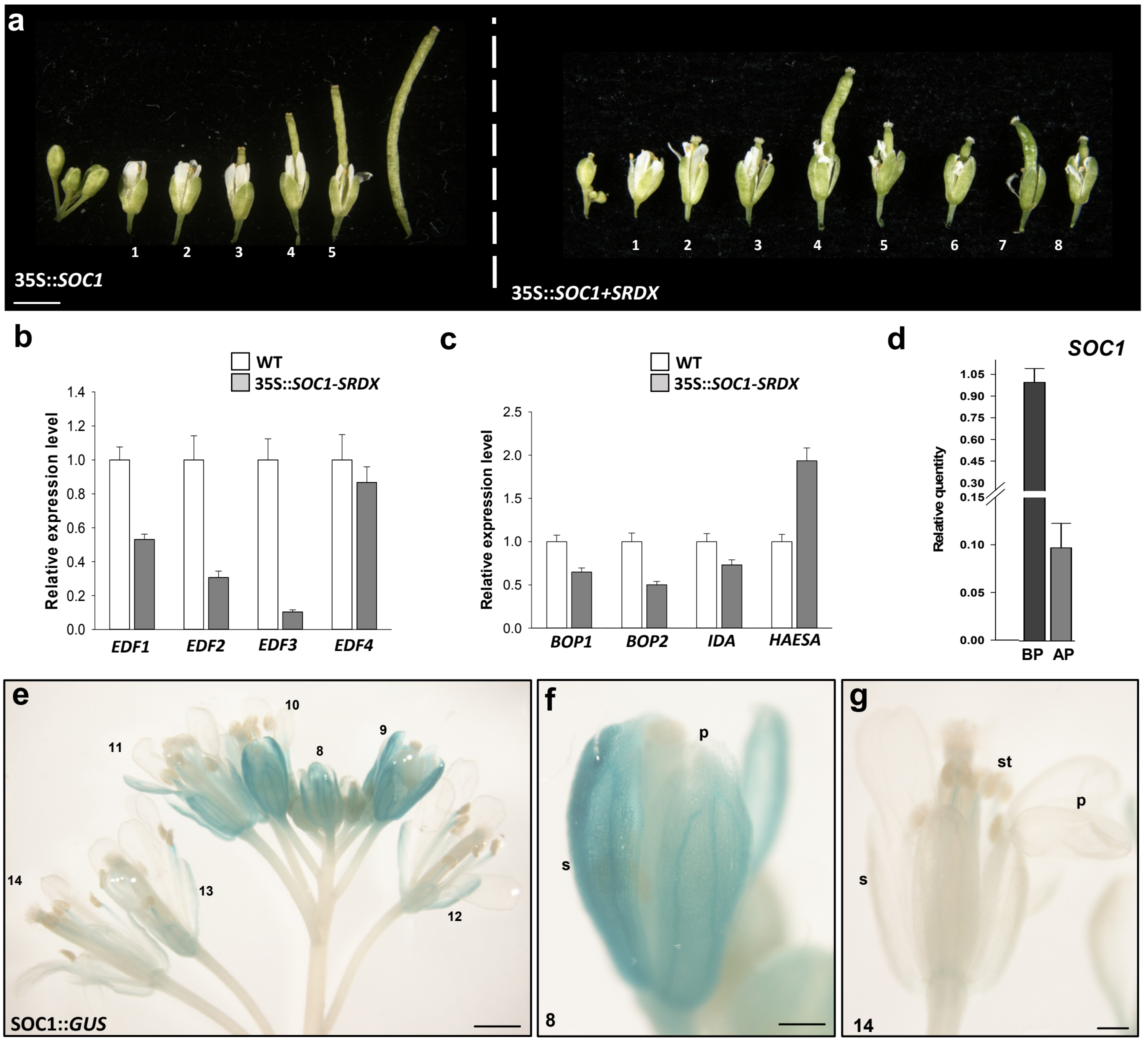

Supplementary Fig. 8 Characterization of the *SOC1* gene through transgenic plants and gene expression analysis in *Arabidopsis*.

a Flowers along the inflorescences of 35S::*SOC1* (left) and 35S::*SOC1+SRDX* (right) plants. The numbers indicate the positions of the flowers. Bar = 2 mm.

b-c The detection of *EDF1-4* (**b**), *BOP1/2*, *IDA* and *HAESA* (**c**) expression in 35S::*SOC1+SRDX Arabidopsis.* Error bars show ± SD. n=1 biologically independent sample. The expression of each gene in the transgenic plants is given relative to that of the wild-type plant, which was set at 1.

d The detection of *SOC1* expression before (BP) and after (AF) pollination.

e GUS was stained in the sepal/petal of flowers of SOC1::*GUS Arabidopsis*. The GUS staining gradually decreased in the mature flowers after stage 12 during the late stage of flower development. The numbers indicate the different developmental stages of *Arabidopsis* flowers. Bar = 1 mm.

f-g Magnified view of stage 8 **(e)** and 14 **(f)** SOC1::*GUS* flowers. GUS was strongly stained in sepal/petal of stage 8 young flower buds and was barely detected in stage 14 mature flowers. s: sepal, p: petal, st: stamen. Bar = 0.2 mm.

Supplementary Fig. 9

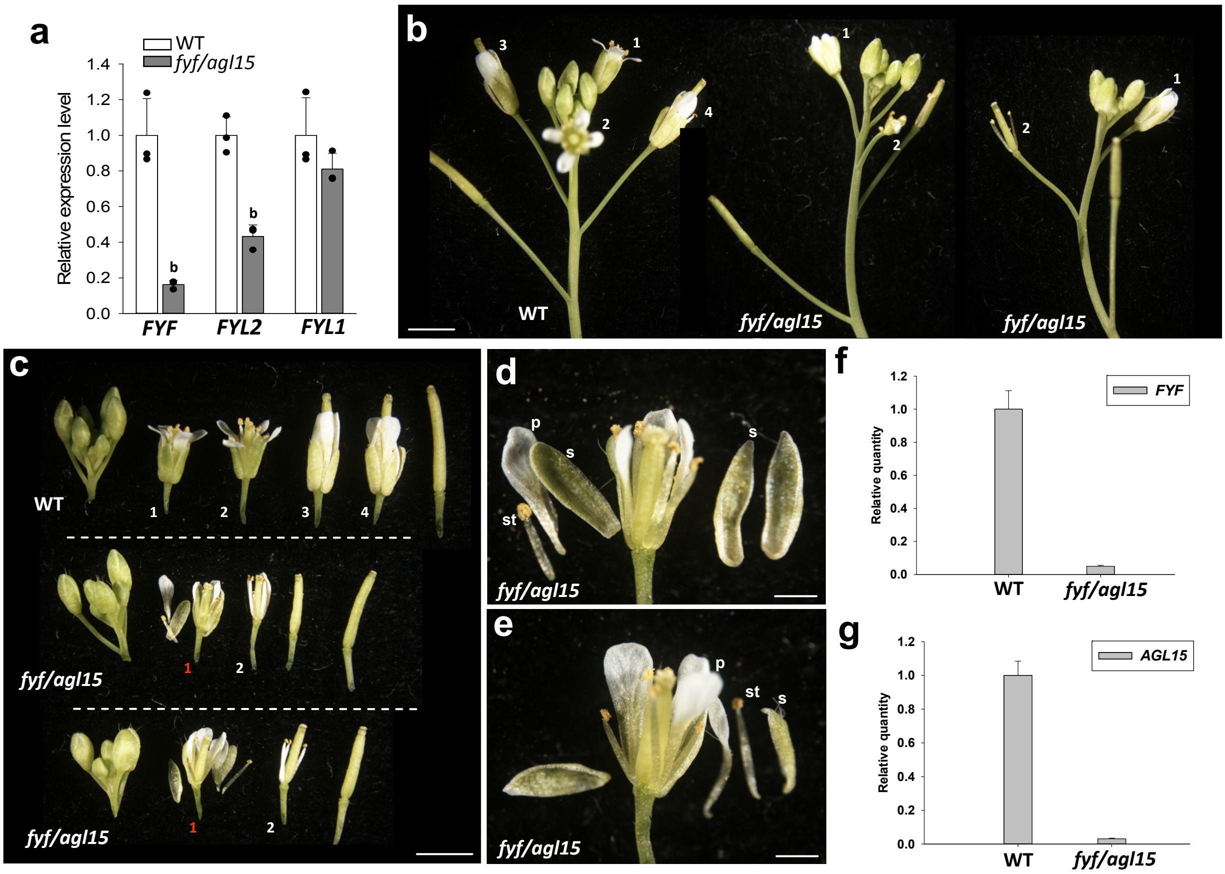

Supplementary Fig. 9 Phenotypic analysis of *fyf/agl15* double mutants.

a Detection of *FYF*, *FYL2* and *FYL1* expression in wild-type (WT) and *fyf/agl15* double mutant *Arabidopsis*. The expression of each gene in the *fyf/agl15* double mutant plants is given relative to that of the wild-type plant, which was set at 1. The letter "b" indicates significant difference from the wild-type (WT) value (b: P<0.01). The two-sided Student's *t*-test was used.

b Flowers along the inflorescence of wild-type (left) and two *fyf/agl15* double mutant (middle and right) *Arabidopsis*. The number indicates the number of open flowers. Bar = 2 mm.

c Detached flowers from the inflorescences of wild-type (top) and *fyf/agl15* double mutant (middle and bottom) *Arabidopsis*. The numbers indicate the positions of the flowers. Bar = 2 mm.

d-e Magnified view of the mature flowers of *fyf/agl15* double mutant *Arabidopsis* from (**b**) clearly showing early senescence and abscission of the flower organs. s: sepal, p: petal, st: stamen. Bar = 0.5 mm.

f-g Detection of *FYF* (**f**) and *AGL15* (**g**) expression in *fyf/agl15* double mutant *Arabidopsis*.

Supplementary Table 1. Oligo nucleotide sequence of primers used in gene cloning and PCR analysis.

Gene name	Primer name	Primer sequence	Restriction site	Use
FYL1 *At5g51870*	FYL1-F	5'- TCTAGAATGGTGAGAGGGAAGATCGAGATCA -3	XbaI	cDNA cloning
	FYL1-R	5'- GGTACCTTATAGCCGAGTCACGGGCAATCCG -3	KpnI	cDNA cloning
	FYL1-ns-R	5'- GGTACCTAGCCGAGTCACGGGCAATCCGAT -3	KpnI	cDNA cloning
	FYL1-P-F	5'-CTGCAGATTTTAGTTAAATTCAAAATGACAAATATATATATTTTGAC-3'	PstI	Promoter cloning
	FYL1-P-R	5'- GGATCCTCTATCTATAATCTTCTCCATCCTG -3'	BamHI	Promoter cloning
	RT-FYL1-F	5'- AGTAGCCGCCATTGTCTTTTCTCAG -3'		Real time PCR
	RT-FYL1-R	5'- TTCACCATTCTATCAATTTCCATCTTCAGC -3'		Real time PCR
FYL2 *At5g51860*	FYL2-F	5'- TCTAGAATGGTGAGAGGAAAGATCGAAATCAAGAAG -3'	XbaI	cDNA cloning
	FYL2-R	5'- GGTACCTTATGGTCGGTTCTTCAGAAATCCAATAAATAG -3'	KpnI	cDNA cloning
	FYL2-ns-R	5'- GGTACCTGGTCGGTTCTTCAGAAATCCAATAAATAGATC -3'	KpnI	cDNA cloning
	FYL2-P-F	5'- CTGCAGCTATGCGAATGCGTCTAATTAATCTG -3'	PstI	Promoter cloning
	FYL2-P-R	5'- GGATCCTCGCTTTATCGTGTTCCTGATAC -3'	BamHI	Promoter cloning
	RT-FYL2-F	5'- GCTAAAGAAGGAAATGGTGACAATGGTG -3'		Real time PCR
	RT-FYL2-R	5'- CTCTATCTGTGTGGCTATCTCTGAAAGTTC -3'		Real time PCR
AGL19 *At4g22950*	AGL19-F	5'- TCTAGAATGGTGAGGGGCAAAACG -3'	XbaI	cDNA cloning
	AGL19-R	5'- GGTACCTTAATTTTGAGGAGGGAATTTTTTGGATTGTC -3'	KpnI	cDNA cloning
	AGL19-ns-R	5'- GGTACCATTTTGAGGAGGGAATTTTTTGGATTGTC -3'	KpnI	cDNA cloning
	AGL19-P-F	5'- AAGCTTAGGAAGCAAGAAAAGTGAGCATG -3'	HindIII	Promoter cloning
	AGL19-P-R	5'- TCTAGAGCGTTCAATTGTTGCTGCTATAC-3'	XbaI	Promoter cloning
	RT-AGL19-F	5'- GTTGAAGGCAGAGGAGAGGA -3'		Real time PCR
	RT-AGL19-R	5'- GTCTTGTCTCAGGAGGTCCA -3'		Real time PCR
SOC1 *At2g45650*	SOC1-F	5'- TCTAGAATGGTGAGGGGCAAAACTC -3'	XbaI	cDNA cloning
	SOC1-R	5'- GGTACCTCACTTTCTTGAAGAACAAGGTAACCCAATG -3'	KpnI	cDNA cloning
	SOC1-ns-R	5'- GGTACCCTTTCTTGAAGAACAAGGTAACCCAATG -3'	KpnI	cDNA cloning
	SOC1-P-F	5'-CTGCAGCATCTGAAATCCAATTAAATTATTATACTATTTGCGTAG-3'	PstI	Promoter cloning
	SOC1-P-R	5'-TCTAGACCTCAGATAACGATCTATGGTATCTTG-3'	XbaI	Promoter cloning
	RT-SOC1-F	5'- GGGATCTCATGAAAGCGAAGT -3'		Real time PCR
	RT-SOC1-R	5'- TCTTGAAGAACAAGGTAACCCA -3'		Real time PCR
AGL14 *At4g11880*	AGL14-F	5'- TCTAGA ATGGTGAGGGGAAAGACAGAG -3'	XbaI	cDNA cloning
	AGL14-R	5'- GGTACC TTAGTTTGAAGGAGGAAACTTTTTGAAGTGTC -3'	KpnI	cDNA cloning
	AGL14-ns-R	5'- GGTACC GTTTGAAGGAGGAAACTTTTTGAAGTGTC -3'	KpnI	cDNA cloning
	AGL14-P-F	5'- GGATCCGAGCGAGAGAGCAAAGAGG-3'	BamHI	Promoter cloning
	AGL14-P-R	5'- CCCGGG TCGCTTTTGATATCTTTCTACTGTTTTAGGTATA -3'	SmaI	Promoter cloning
	RT-AGL14-F	5'- TCACAGCAATCGAAGGACGA-3'		Real time PCR
	RT-AGL14-R	5'- GATGCGTCAAGTCCTTCTCC -3'		Real time PCR
AGL15 *At5g13790*	AGL15-F	5'- TCTAGAATGGGTCGTGGAAAAATCGAG -3'	XbaI	cDNA cloning
	AGL15-R	5'- GGTACCCTAAACAGAGAACCTTTGTCTTTTGGC -3'	KpnI	cDNA cloning
	AGL15-ns-R	5'- GGTACCAACAGAGAACCTTTGTCTTTTGGC -3'	KpnI	cDNA cloning

	AGL15-P-F	5’- CTGCAGGGTGAACAGTTTTATTGAACATGTATGGTG-3’	PstI	Promoter cloning
	AGL15-P-R	5’- GGATCCTTTCCCCCAAAATTGAACAGAGAGAAG-3’	BamHI	Promoter cloning
	RT-AGL15-F	5'- GAGAAAGAGAGAGCCCGTCA-3'		Real time PCR
	RT-AGL15-R	5'- CCTCTTTCTGCAGTTTCGCT -3'		Real time PCR
AGL6	AGL6-F	5'-TCTAGAATGGGAAGAGGGAGAGTGG-3'	XbaI	cDNA cloning
At2g45650	AGL6-R	5'-GGTACCTCAAAGAACCCAACCTTGGACG-3'	KpnI	cDNA cloning
	AGL6-ns-R	5'-GGTACCAAGAACCCAACCTTGGACG-3'	KpnI	cDNA cloning
	AGL6-P-F	5'-GTCGACGTTGTTGGTTAACGCCTCTCGGAG-3	SalI	Promoter cloning
	AGL6-P-R	5'-CCCGGGAAGAACCCAACCTTGGACGAAATTAG-3'	SmaI	Promoter cloning
	RT-AGL6-F	5'-GAGCCTTCTCATCCTAATGTATTG-3'		Real time PCR
	RT-AGL6-R	5'-CGTAGTAATGTTGTTGAAACCCTA-3'		Real time PCR
EDF1	RT-EDF1-1	5'-TCACAAACACAACAAATATGGAATACAG-3'		Real time PCR
At1g25560	RT-EDF1-2	5'-GCTTTGGAGTAGTAGAGATGGAGAG-3'		Real time PCR
EDF2	RT-EDF2-1	5'-CTAAACAACACGCCGAGAAACAC-3		Real time PCR
At1g68840	RT-EDF2-2	5'-GCCGAACCACCACCTGAACC-3'		Real time PCR
EDF3	RT-EDF3-1	5'-ACACATATACATTCACATTACTAATCTC-3'		Real time PCR
At3g25730	RT-EDF3-2	5'-AATGGAATCTGTAGTTGTAGAGC-3'		Real time PCR
EDF4	RT-EDF4-1	5'-CCGAAACATCACGCAGAGAAAC-3'		Real time PCR
At1g13260	RT-EDF4-2	5'-AACCCGACCCGCATCTAAATC-3'		Real time PCR
BOP1	RT-BOP1-1	5'-CGAAGAAGGAAACAATAACAGTAACGATAA-3'		Real time PCR
At3G57130	RT-BOP1-2	5'-GTCATCTCCAATATCACGATTTGTAGCA-3'		Real time PCR
BOP2	RT-BOP2-1	5'-GCAACAACCAAAACAATGATAACAATACC-3'		Real time PCR
At2G41370	RT-BOP2-2	5'-CTGGACCCATCTGACCCGTACCT-3'		Real time PCR
IDA	IDA-3	5'-GGATCCTTTTCAATTTTGTTATTGCAT -3'	BamHI	Promoter cloning
At1G68765	IDA-4	5'- CATATGGTAGTCAATGTTTTTTTCTTCTCTTT -3'	NdeI	Promoter cloning
	RT-IDA-1	5'-CATTCATTTACTCAAAGAAGTGGAAGTG-3'		Real time PCR
	RT-IDA-2	5'-GTGTCTCTTAGAAGGAGCAGAAGG-3'		Real time PCR
HAESA	RT-HAESA-1	5'-TGATTCAGAACTTGGAGATAAAGATATGG-3'		Real time PCR
At4G28490	RT-HAESA-2	5'-CTTCCGTGTAGTAAGGCGAGAG-3'		Real time PCR
ERF1	RT-ERF1-1	5'-GAGCAGTCCACGCAACAAAC-3'		Real time PCR
At3G23240	RT-ERF1-2	5'-TCTCCGAAAGCGACTCTTGAAC-3'		Real time PCR
UBQ10	RT-UBQ10-1	5'-CTCAGGCTCCGTGGTGGTATG-3'		Real time PCR
At4G05320	RT-UBQ10-2	5'-GTGATAGTTTTCCCAGTCAACGTC-3'		Real time PCR
FYF	AGL42-1	5'-GGATCCATTCTAGGGCTCCTGACAGA-3'	BamHI	cDNA cloning
At5g62165	AGL42-2	5'-GGGATCCTCGCAGTTTCTATTTGGC-3	BamHI	cDNA cloning
	RT-AGL42-1	5'-AGCAATCACGACTCACAAATTCAC-3'		Real time PCR
	RT-AGL42-2	5'-AGCCTTTCTTTCTCGGACCTTTC-3'		Real time PCR
SAG12	RT-SAG12-1	5'-TCCAATTCTATTCGTCTGGTGTGT-3		Real time PCR
At5G45890	RT-SAG12-2	5'-CCACTTTCTCCCCATTTTGTTC-3		Real time PCR
