# Supplementary material for: Regulatory network for FOREVER YOUNG FLOWER-like genes in regulating Arabidopsis flower senescence and abscission
Source: Commun Biol. 2022 Jul 5;5:662. doi: 10.1038/s42003-022-03629-w (PMC9256709; doi:10.1038/s42003-022-03629-w)
Supplement: Supplementary file 2 — Description of Additional Supplementary Files [file 42003_2022_3629_MOESM2_ESM.pdf]

Description of Additional Supplementary Files

File name: Supplementary Data 1
Description: The source data behind the graphs and charts in the paper
